# Temperament multi-trajectory groups across adolescence: Associations with adulthood psychopathology and polygenic scores in TRAILS

**DOI:** 10.1017/S0954579425100680

**Published:** 2025-09-17

**Authors:** Frances L. Wang, Shirley Duong, Heather M. Joseph, Traci M. Kennedy, Catharina Hartman

**Affiliations:** 1 Department of Psychiatry, School of Medicine, University of Pittsburgh, Pittsburgh, USA; 2 University Medical Center Groningen, Department of Psychiatry, Interdisciplinary Center Psychopathology and Emotion regulation, University of Groningen, Groningen, The Netherlands

**Keywords:** Externalizing, internalizing, polygenic risk score, temperament multi-trajectory groups

## Abstract

It is well-established that adolescents’ temperament trajectories predict future psychopathology. Less well understood is how temperament traits co-develop from adolescence to young adulthood. We characterized how youths’ trajectories of effortful control, frustration, affiliation, and shyness formed multi-trajectory groups and examined their associations with adulthood psychopathology and polygenic risk scores (PRS). Participants were drawn from a larger longitudinal cohort (*N* = 1412). Effortful control, frustration, affiliation, and shyness were measured four times from ages 10-23. Adulthood internalizing and externalizing problems were measured at ages 24–27. PRS for externalizing problems and major depressive disorder were calculated. Group-based multi-trajectory analyses showed that a five-group model fit best, including “*high-risk”* on all temperament traits, *“undercontrolled”* and exuberant, *“low-risk”* on all traits, *“overcontrolled”* and inhibited, and *“low affiliation”* groups that differed on both the levels and slopes of temperament traits over time. The *undercontrolled* group showed the highest, and *overcontrolled* the lowest, externalizing PRS scores. The *high-risk* group showed heightened scores on the depression PRS. We found specific linkages between the *high-risk* group and withdrawn/depressed symptoms and the *high-risk* and *undercontrolled* groups with externalizing problems. Findings shed light on developmental patterns of temperament in adolescence-to-adulthood and unique combinations of temperament trajectories with specific linkages to etiologic factors and psychopathology.

## Introduction

Temperament traits have been defined as heritable individual differences in reactivity and regulation (Rothbart & Ahadi, 1994). How temperament traits develop from childhood to adolescence has been shown to be important in the prediction of psychopathology in adulthood (Lawson et al., 2023). However, it is less well understood how temperament traits *co-develop* alongside one another from adolescence to young adulthood. Moreover, little is known about whether individuals with distinct profiles of temperament co-development differ on mental health outcomes and genetic risk factors. Holistically characterizing individuals’ profiles of temperament trajectories from adolescence to adulthood based on the most commonly occurring patterns over time could yield insights into pathways to psychopathology. This research could also clarify existing theories about patterns of temperament development across the sensitive period of adolescence. Thus, in this study we aimed to characterize trajectory groups underlying five dimensions of temperament from adolescence to young adulthood. We also aimed to examine how these temperament trajectory groups were related to polygenic risk scores as well as to future psychopathology.

### Development of temperament traits from adolescence to young adulthood

One of the most widely studied models of temperament in childhood is defined by three superordinate dimensions of temperament (Rothbart et al., 2001). The first, effortful control, has been defined as the ability to suppress a dominant response to perform a subdominant response. It is comprised of three lower-order facets including attentional, inhibitory, and activational control. The second, negative emotionality, describes an individual’s propensity to experience negative emotions such as frustration and fear. The third, positive emotionality, is defined as the propensity to experience positive emotions and includes facets like affiliation and high-intensity pleasure. Notably, shyness is another lower-order temperament facet that has been grouped with negative emotionality in some studies (Capaldi & Rothbart, 1992) and with positive emotionality in others (Ellis & Rothbart, 2001). Temperament traits are also conceptually similar to personality traits, which are commonly studied using the Big Five Model. These personality traits include conscientiousness, neuroticism, extraversion, agreeableness, and openness to experience (Costa & McCrae, 1992). Effortful control has been shown to be conceptually and empirically related to the Big Five personality trait of conscientiousness; negative emotionality to neuroticism; the high-intensity pleasure facet of positive emotionality to extraversion; and the affiliation facet of positive emotionality to agreeableness (Victor et al., 2016). Notably, despite originating from different models, researchers have not found compelling conceptual or empirical distinctions between temperament and personality (Clark & Watson, 2008; Lawson et al., 2023; Shiner et al., 2021).

Temperament and personality traits show meaningful patterns of change from late childhood to early adulthood. Research on effortful control and conscientiousness have largely shown decreases in this trait across adolescence (Denissen et al., 2013), with increases emerging in adulthood (Bleidorn et al., 2022). This pattern is in line with the disruption hypothesis of personality change, which posits that biological, social, and psychological shifts from childhood to adolescence cause temporary dips in certain aspects of personality maturation (Soto & Tackett, 2015). Conversely, the bulk of research on negative emotionality has shown that this trait, and its facets, tended to decrease over adolescence (Denissen et al., 2013; Soto et al., 2011) and adulthood (Bleidorn et al., 2022), consistent with the maturity principle; that is, individuals become more regulated, prosocial, and emotionally stable over time (Bleidorn et al., 2013). Finally, research on surgency-related traits tended to be less consistent, especially in adolescence. Research variably showed that surgency and affiliation increased (Laceulle et al., 2012; Zohar et al., 2019), decreased (Akker et al., 2010; Brandes et al., 2021), or showed no change (Borghuis et al., 2017; De Fruyt et al., 2006) in adolescence. In contrast, a meta-analysis converged on findings that extraversion (i.e., personality analogue of surgency) stayed relatively stable into adulthood and that agreeableness (i.e., analogue of affiliation) increased in adulthood (Bleidorn et al., 2022).

Although understanding normative developmental trends in temperament and personality development are important, there is also substantial variability in how temperament develops over time (Borghuis et al., 2017). This suggests that some individuals mature, whereas others experience disruptions, in temperament development across adolescence and young adulthood. The existence of meaningful subgroups of individuals who show differing trajectory patterns across multiple temperament traits over time would explain inconsistencies in existing research focused on the *means* of temperament trajectories. This supposition is further supported by research showing that infants belonging to trajectory groups characterized by higher negative emotionality over time tended to also belong to trajectory groups characterized by low regulatory ability over time (Giesbrecht et al., 2022), and the same for low positive emotionality and regulatory ability. However, few studies have examined co-occurring temperament trajectories including more than two traits and that focused adolescence to early adulthood. This study characterized unique groups varying in their longitudinal trajectories on *multiple* temperament dimensions (i.e., effortful control, frustration, affiliation, and shyness) spanning late childhood through young adulthood using group-based multi-trajectory analysis (Nagin et al., 2018). Use of four assessment points allowed linear and quadratic slopes to be fit to temperament trajectories, providing insights into maturation (linear slopes) vs. disruption hypotheses (quadratic slope).

### Temperament multi-trajectory groups and future psychopathology

Research has long established a relationship between individual differences in temperament and the development of future psychopathology (Caspi et al., 1996). The early-appearing and predictive nature of temperament dimensions make them important for understanding which children are most at risk for certain types of psychopathology. Generally, research has shown that poor effortful control and negative emotionality (and their lower-order dimensions) are broad, transdiagnostic predictors for many different types of psychopathology spanning internalizing and externalizing problems, such as depressive and anxiety symptoms, rule-breaking and aggressive behaviors, and attentional problems (Hankin et al., 2017; Oldehinkel et al., 2004; Santens et al., 2020; Wang et al., 2016). Moreover, positive emotionality has been shown to be directionally linked with internalizing versus externalizing problems, such that higher positive emotionality and related facets are linked with greater externalizing problems whereas lower positive emotionality is linked with greater internalizing problems (Hankin et al., 2017; Oldehinkel et al., 2004; Ormel et al., 2005).

Temperament trajectories, rather than just their relative levels, may also be important in determining later psychopathology. Indeed, one’s temperamental disposition could increase difficulties in coping with the “storm and stress” of adolescence, cause selection into maladaptive peer groups, or evoke stressful environmental circumstances (Hopwood et al., 2022; Klein et al., 2011; Lawson et al., 2023). This process may be reflected as more disruption or less maturation in temperament traits over time, leading to worsened mental health in adulthood. Accordingly, research showed that decreases in effortful control and increases in irritability and fear from ages 8–12 to 11–15 predicted greater levels of both internalizing and externalizing problems at ages 11–15 (Lengua, 2006). Another study found that decreases in extraversion from 8 to 14 years predicted adolescents’ internalizing problems, decreases in conscientiousness predicted externalizing problems, and decreases in emotional stability predicted both types of problems (van den Akker et al., 2010). Using the same sample as the current study, Laceulle et al. (2014) found that changes in frustration across two time points (11–16 years old) predicted both internalizing and externalizing disorders at ages 16–19, whereas changes in fear predicted internalizing but not externalizing disorders (and no effect of changes in high-intensity pleasure, shyness, affiliation, or effortful control). Despite studying similar ages, across all studies the only consistent finding was that changes in frustration and related variables (i.e., irritability, emotional stability) predicted both internalizing and externalizing problems, whereas variable findings were reported for all other facets of temperament change.

Characterizing co-development of multiple temperament traits (i.e., temperament multi-trajectory groups) could provide more specificity and clarity in the prediction of future psychopathology than constituent temperament traits alone. Indeed, prior research showed the importance of interactions between temperament traits in predicting risk for future psychopathology (e.g., combination of low effortful control and high negative emotionality; Oldehinkel et al., 2007; Phillips et al., 2022; Van Beveren et al., 2019). As testing interactions between temperament traits, especially more than two, requires large sample sizes and can become unwieldy to interpret, the multi-trajectory group approach is a helpful way to understand these interactive processes among multiple temperament *trajectories* while allowing each trajectory to assume any shape (i.e., linear, quadratic; increasing, decreasing).

Moreover, similar cross-sectional studies that examined multi-trait profiles of temperament in relation to future psychopathology yielded somewhat more specificity in these predictions. Common themes that emerged from this area of research were that a “well-adjusted” group of youths with adaptive temperament functioning across domains typically showed lower risk for internalizing and externalizing problems; a “dysregulated” group (low effortful control-related traits and high negative emotionality- and surgency-related traits) showed risk for externalizing and internalizing problems; and a group characterized by higher negative affectivity but lower surgency-related traits showed risk for internalizing problems (Caspi et al., 1996; Moreira et al., 2021; Rettew et al., 2008; Robins et al., 1996). Thus, multi-trait profiles of temperament can offer an understanding of the “whole person” and how a confluence of traits leads to specific psychopathology outcomes.

### Temperament multi-trajectory groups and polygenic risk scores

Polygenic risk scores (PRS) represent the weighted sum of measured genetic variants derived from existing genome-wide association studies (GWAS) on certain phenotypes (Purcell et al., 2009). A robust literature has emerged to characterize the nomological network of PRS, i.e., what PRS capture across various stages of development, to understand whether and how genetic risk for different forms of psychopathology manifests before the onset of disorder. Interestingly, temperament/personality dimensions and trajectories are moderately to highly heritable in twin studies of youth and adults (Bleidorn et al., 2009; Liu et al., 2023; Saudino, 2009) and share moderate-to-high genetic influences with internalizing and externalizing problems (Lo et al., 2017; Tackett et al., 2011, 2013). Accordingly, temperament dimensions and their trajectories have been shown to be predicted by PRS indexing various internalizing and externalizing problems. For example, a PRS indexing smoking predicted behavioral disinhibition in adolescence (Hicks et al., 2021), a PRS of cannabis use predicted a measure of risk liability for substance use disorder (Brick et al., 2021), and a PRS of depression predicted neuroticism (Navrady et al., 2018) and trajectories of trait persistence in adulthood (Lavonius et al., 2024).

Interestingly, research showed that latent *profiles* of temperament traits in adolescence were modestly to highly heritable (0.16-0.80; (Murillo et al., 2024), in line with prominent theories that temperament *types* are biologically based (Kagan, 2018). Thus, it would be of interest to understand how PRS for major forms of psychopathology relate to multi-trajectory temperament groups that holistically describe individuals’ temperament functioning. We examined how PRS indexing genetic risk for internalizing or externalizing problems predicted temperament multi-trajectory groups and adulthood psychopathology. PRS for broadband internalizing and externalizing problems were specifically examined because genetic influences underlying various internalizing or externalizing problems, respectively, are largely shared (Grotzinger et al., 2022; Schwaba et al., 2025) and to reduce the number of tests performed. We created a PRS based on a large meta-GWAS for externalizing problems (Linnér et al., 2021), which was well powered to detect genetic effects. Because similarly large GWAS for broad internalizing problems were not available (e.g., Jami et al., 2022), we created a PRS based on a large meta-GWAS for major depressive disorder, as this phenotype can act as a well-powered proxy for broader internalizing problems given very high genetic intercorrelations (Grotzinger et al., 2022; Schwaba et al., 2025).

### Current study

We used group-based multi-trajectory modeling to derive groups based on similarities in their trajectories of effortful control, frustration, affiliation, and shyness across four assessment waves spanning ages 10 – 23 in a large Dutch sample. These four temperament dimensions were chosen because they broadly represent commonly defined facets of temperament and were measured at four waves in this sample. We hypothesized that we would find multiple unique groups differing on their levels and slopes of temperament trajectories, with at least one group representing the highest risk on all temperament traits and one representing the lowest risk on all temperament traits. Next, we examined how PRS for major depressive disorder and externalizing problems predicted the temperament multi-trajectory groups over and above relevant covariates. Finally, we examined whether temperament multi-trajectory groups prospectively predicted young adulthood psychopathology both with and without controlling for earlier levels of psychopathology (i.e., depressive symptoms, anxiety symptoms, delinquency, aggressive behaviors, and attentional problems). Regression models included as covariates ancestry principal components, sex, parental psychopathology, and SES. Indeed, these variables play an important role in the development of temperament and later psychopathology and it is useful to understand if the PRS and/or temperament trajectory groups remain important predictors after controlling their effects. We hypothesized that temperament trajectory groups would differ in their levels of PRS and adulthood psychopathology. We also expected that the highest-risk temperament trajectory group would show the highest risk on at least one PRS and on several internalizing and externalizing outcomes relative to the lowest-risk temperament trajectory group. Moreover, we hypothesized that some trajectory groups with relatively lower risk on temperament traits could nonetheless show greater risk for certain PRS or types of psychopathology relative to the highest-risk temperament trajectory group.

## Method

We report how we determined our sample size all data exclusions, manipulations, and measures in the study in the following sections. This study was not preregistered.

### Participants

The TRacking Adolescents’ Individual Lives Survey (TRAILS) is a prospective cohort of Dutch adolescents followed bi- or tri-annually from 11 to at least 30 years (Huisman et al., 2008; Oldehinkel et al., 2014). This study uses data from six assessment waves (T1–T6). Children born between October 1, 1989, and September 30, 1991, were eligible, provided they met inclusion criteria and their schools were willing to participate. Over 90% of the schools enrolling 2,935 eligible children agreed to participate. Seventy-six percent of these children and their parents consented to participate (T1, *N* = 2,230, mean age(*SD*) = 11.1(0.6) years, 50.8% girls). Subsequent waves had good retention (T2 *M*_age_ = 13.6, 96%; T3 *M*_age_ = 16.3, 81%; T4 *M*_age_ = 19.1, 84%; T5 *M*_age_ = 22.3, 80%; T6 *M*_age_ = 25.7, 73%).

Non-response and attrition during follow-ups were somewhat higher in males and in adolescents of non-Western ethnicity with divorced parents, low parental socio-economic status, low intelligence quotient and academic achievement and poor physical health and with behavior and substance use problems. Non-response showed little to no association with urbanization, parental religiousness, being an only child, or recent self-reports of anxiety and mood problems (Nederhof et al., 2012; Oldehinkel et al., 2014). All assessments were approved by the national ethical committee (CCMO, www.ccmo.nl; P00.0246C, 02-08-2000; P00.0246C, 25-10-2000; P03.105C,03-05-2005; P03.105C – dd 28-09-2006; NL22114.042.08; 27-10-2008; NL22114.042.08. 21-09-2009; NL38237.042.11, 07-02-2012) and all procedures were performed in compliance with relevant laws and institutional guidelines. TRAILS data are not open source but accessible for researchers outside the TRAILS consortium by submitting a publication proposal (www.trails.nl/en/home).

Participants were included in the study if they had genomic information (*N* = 1842) and if they passed genetic quality control procedures (see below) and were of European ancestry (*N* = 1412). Included versus excluded participants did not significantly differ in anxious/depressed symptoms, or assigned sex. Included participants showed lower withdrawal/depression (*t*(956.16) = 2.04, *p* < .05), aggression (*t*(934.81) = 3.57, *p* < .001), delinquency (*t*(922.64) = 2.57, *p* < .05), attention problems (*t*(989.93) = 2.03, *p* < .05), and higher SES (*t*(2683.8) = -13.39, *p* < .001), and lower likelihoods of parental depression (*X* ^*2*^ = 9.96, *p* < .01), anxiety (*X* ^*2*^ = 7.22, *p* < .01), substance use (*X* ^*2*^ = 32.78, *p* < .001), and antisocial personality (*X* ^*2*^ = 36.39, *p* < .001). These small differences were likely detectable due to our large sample and are of little substantive significance (see Figures S1-S3, Supplement; Sharma, 2021).

### Genotyping

At T3, adolescents provided blood samples. Participants were genotyped using the Golden Gate Illumina BeadStation 500 platform and the HumanCytoSNP-12 BeadChip (Illumina Inc., San Diego, CA, USA). Genotypic data were merged, overlapping single nucleotides (SNPs) checked for concordance, and imputed against the 1000 Genomes Project Phase 3 global reference panel. Quality control was conducted using PLINK v1.07 and v1.9 (Chang et al., 2015; Purcell et al., 2007). SNPs with call rate below 95%, a minor allele frequency (MAF) below .05, missingness rates above 5%, and a Hardy–Weinberg disequilibrium *p* value < 0.0005 were excluded. Individuals with more than 5% missingness on SNP data and who were of non-European ancestry were excluded (*N* = 430). To prevent familial clustering of effects, we excluded one of each pair of related individuals (closer than third degree). Alleles were aligned with 1,000 Genomes, excluding SNPs with MAFs deviating > 0.15 from the reference set. These procedures resulted in *N* = 7,781,794 SNPs and *N* = 1412 participants.

### Measures

#### Temperament

Temperament was assessed by the parent-report version of the Early Adolescent Temperament Questionnaire-Revised (EATQ-R) assessed at waves T1, T3, T4, and T5 (Putnam et al., 2001). We included affiliation (“My child finds it important to have a good relationship with others”), effortful control (e.g., “It takes a lot of effort for my child to get things done on time”), frustration (“My child is annoyed by little things that other children do”), and shyness (“My child is shy when they meet new people”), as these were the temperament dimensions measured at all four waves in this longitudinal cohort. Each item was rated on a five-point scale (1 = hardly ever true; 5 = almost always true). Items used in the analyses were informed by previous work on the internal factor structure of these data (Oldehinkel et al., 2004) and whether they were administered at all four assessments. This resulted in four affiliation items, seven effortful control items, four frustration items, and two shyness items (Table S1). Across T1, T3, T4, and T5, Cronbach’s alpha ranged from 0.85–0.87 for effortful control, 0.68–0.74 for affiliation, 0.72–0.77 for frustration, and 0.67–0.75 for shyness.

#### Internalizing and externalizing symptoms

Participants reported on internalizing and externalizing symptoms during the last 6 months using the Adult Self Report (ASR; Achenbach & Rescorla, 2003) at T6. The ASR covers a broad range of emotional and behavioral problems (‘0 = not true’, ‘1 = somewhat or sometimes true’, ‘2 = very or often true’). We used the syndrome scales of withdrawn/depressed (9 items; Cronbach’s *α* = 0.80), anxious/depressed (18 items; *α* = 0.93), attentional problems (15 items; *α* = 0.86), delinquency (including substance use items; 14 items; *α* = 0.75), and aggression (15 items; *α* = 0.84). We also used the same syndrome scales at T1–T3 (averaged) as controls for earlier psychopathology to test whether temperament trajectory groups predicted later psychopathology above and beyond stability in psychopathology over time.

#### Polygenic risk scores

The PRS were based on summary statistics obtained from two separate meta-GWAS. For major depressive disorder, the meta-GWAS examined proxy phenotypes such as affirmative responses to “Have you ever seen a psychiatrist for nerves, anxiety, tension, or depression?” and other clinically derived phenotypes for major depressive disorder (Howard et al., 2019). The inclusion of broad definitions of depression is in line with our goals of using this PRS as a proxy for internalizing problems. As this GWAS For externalizing problems, Linnér et al. (2021) conducted genomic structural equation modeling to model a latent factor underlying several externalizing problems (i.e., ADHD, alcohol use, cannabis use, smoking initiation, general risk tolerance, number of sexual partners) and performed a GWAS on the externalizing factor.

GWAS summary statistics derived from European participants (*N*_*Depression*_ = 500,199; *N*_*Externalizing*_ = 1,045,957) were used to calculate a depression and externalizing PRS within TRAILS participants genetically confirmed to be European. We used PRS-CS (Ge et al., 2019) to calculate the posterior effect sizes of variants by using a Bayesian regression framework and continuous shrinkage priors. PRS-CS models local linkage disequilibrium (LD) patterns and excludes variants with small effects, thereby precluding the need for LD pruning and *p*-value thresholding. As PRS-CS requires an external LD reference panel, we selected European samples from the 1000 Genomes project (phase 3, NCBI GRCh37). Posterior effect sizes were estimated for SNPs by PRS-CS using the –score function in PLINK (Purcell et al., 2007).

#### Covariates

Participants reported on their “biological sex” at baseline (0 = female, 1 = male; hereafter referred to as sex assigned at birth). Lifetime presence of parental substance use, depression, antisocial behavior, and anxiety were measured with the TRAILS Family History Interview (Ormel et al., 2005) for both parents, based on a single informant. Each condition was introduced by a vignette describing DSM-IV characteristics (available on request). Parental psychopathology was defined as having at least one parent with the condition (1= present; 0 = absent). SES was calculated per family at T1 as a combination of fathers’ and mothers’ education (1 = elementary to 5 = university), occupation (nine categories based on the International Standard Classification of Occupations; Ganzeboom & Treiman, 1996), and monthly household income, or the available data for single-parent households. The SES composite was created by averaging *z*-scores of available variables. Ancestry principal components (PCs) were also computed based on genomic data (Campbell et al., 2005).

### Analytic plan

#### Missing data

Cases were excluded based on those missing PRS scores. Within that group, patterns of missing data were examined for internalizing and externalizing outcomes (22%), SES (7%), and parental psychopathologies (1%). Missing data for those variables (but not for PRS) were imputed using multiple imputation by chained equations (MICE) with the ‘mice’ package in R (van Buuren & Groothuis-Oudshoorn, 2011). Forty datasets were created in which missing data were estimated using other variables in the dataset, excluding those that need to be imputed, in a series of predictive models. The imputation model included all analysis variables (i.e., PRS, internalizing and externalizing outcomes, regression covariates), and predictive mean matching, which is robust to integer and skewed data, was used to select the imputed values. This method selects an observation from the original, non-missing data that has a predicted or expected value close to the predicted value of the missing sample. Regression analyses were conducted using all 40 imputed datasets and model estimates were pooled by averaging.

#### Measurement invariance

To determine that the same constructs were captured over time for longitudinal analyses and across sex, we tested whether affiliation, effortful control, frustration, and shyness showed measurement invariance across the four assessments and, separately, by sex. Increasingly constrained multi-group confirmatory factor models using full-information maximum likelihood estimation were estimated for each outcome in Mplus v.8 (Muthén & Muthén, 1998). Model fit at each stage of invariance testing was assessed based on RMSEA < .06, SRMR < .08, CFI > .90, TLI > .90, changes in RMSEA < .015, changes in CFI < .01, and lower BIC (Putnick & Bornstein, 2016). Table S2 summarizes results of invariance testing. Full measurement invariance was found for all temperament dimensions by sex (scalar invariance with acceptable fit). Next, for measurement invariance by assessment period (T1, T3, T4, and T5), full measurement invariance for affiliation items was found. After freeing one item each, acceptable fit was achieved in the partial scalar invariance models for effortful control and frustration. Given that shyness was made up of two items, invariance was assumed after estimating a model with factor loadings and intercepts constrained to be equal and evaluating global model fit. This model showed acceptable fit. Overall, all temperament outcomes showed invariance across time. Factor scores from these models were saved for use in group-based multi-trajectory models.

#### Temperament multi-trajectory modeling

Group-based multi-trajectory modeling was conducted using Stata (Version 17 SE) and the traj command (Jones & Nagin, 2013). Affiliation, effortful control, frustration, and shyness measured at four waves were modeled following censored normal distributions and age was treated as an integer. Models for up to six independent multi-trajectory groups were estimated using stepwise elimination of non-significant polynomials.

The final model was selected using the following fit indicators: the largest Bayesian Information Criterion (BIC), the group(s) at which change in BIC plateaued, the odds of correct classification (minimum of 5), the average posterior probability of group membership (minimum of .70), standard deviation of group membership probabilities (smaller values), and entropy (values closer to 1 indicating better classification accuracy). As an increasing number of multi-trajectory groups tends to always result in “better” fit, we also evaluated models based on substantive interest (Nagin et al., 2018).

#### Prediction models

Multinomial logistic regression was used to predict membership in each temperament multi-trajectory group using PRS and all covariates (sex assigned at birth, parental depression, anxiety, substance use, antisocial behavior, SES, and 10 ancestry PCs). A series of multiple linear regression models were estimated to predict psychopathology outcomes. Predictors included PRS, temperament multi-trajectory groups, and covariates (sex assigned at birth, parental depression, anxiety, substance use, antisocial behavior, SES, and 10 ancestry PCs). We tested models with PRS simultaneously. Primary variables (i.e., temperament trajectory groups and PRS) in models predicting psychopathology were adjusted for multiple testing using the false-discovery rate method, which calculates adjusted *p*-values, or *q*-values (Benjamini & Hochberg, 1995). Finally, models predicting psychopathology outcomes were tested both with and without controlling for prior levels of psychopathology (average of T1–T3 scores).

## Results

### Descriptive statistics

Table 1 shows descriptive statistics for continuous variables. 48% were assigned female at birth. Lifetime presence of parental psychopathologies in descending order were depression (36.96%), anxiety (20.94%), antisocial behavior (8.22%), and substance use (8.14%).

**Table 1.** Descriptive statistics of PRS and psychopathology outcomes (non-imputed data)

	*n*	Mean	SD	Median	Range	Skew	Kurtosis	SE
**PRS**								
Depression	1412	−.004	0.11	−.005	−.40 – .32	−0.03	0.05	.003
Externalizing	1412	−.004	0.08	−.003	−.25 – .25	0.04	−0.02	.001
**Internalizing**								
Withdrawal	1099	0.29	0.32	0.222	0 – 1.78	1.485	2.278	0.01
Anxiety	1099	0.50	0.43	0.429	0 – 2	0.942	0.422	0.01
**Externalizing**								
Aggressive behavior	1099	0.24	0.25	0.133	0 – 1.40	1.260	1.304	0.01
Delinquent behavior	1099	0.16	0.19	0.071	0 – 1.14	1.982	5.034	0.01
Attention problems	1099	0.46	0.34	0.400	0 – 1.67	0.943	0.626	0.01
**Age in years at each time point**								
T1 (Age 11)	2017	11.10	0.55	11.01	10.01 – 12.58	0.47	2.55	0.01
T3 (Age 16)	1844	16.19	0.65	16.10	15 – 17.99	0.67	2.97	0.02
T4 (Age 19)	1995	19.08	0.63	19.01	18.03 – 20.99	0.61	2.84	0.01
T5 (Age 22)	1920	22.21	0.65	22.15	21 – 23.99	0.38	2.42	0.01
**Socioeconomic status**	1406	0.14	0.74	0.15	−1.89 – 1.74	−0.14	−0.74	0.02

*Note.* Socioeconomic status is an average of *z*-scores.

### Zero-order correlations

**Figure 1.**
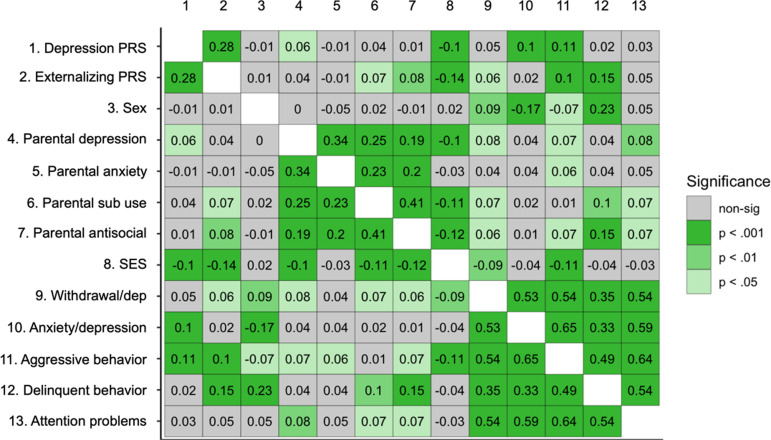
Heatmap of zero-order correlations of the PRS and psychopathology outcomes (imputed data).

Correlations among all study variables except temperament multi-trajectory groups are shown in Figure 1. Higher levels of the depression PRS were correlated with the externalizing PRS, parental depression, SES, anxiety/depression, and aggressive behavior. Higher levels of the externalizing PRS were correlated with parental antisocial behavior and substance use, SES, withdrawal/depression, aggression, and delinquency.

Correlations between temperament outcomes at each time point and PRS were examined (Table S3). At all assessments, higher levels of the depression PRS were correlated with lower effortful control and higher frustration. Higher levels of externalizing PRS were correlated with lower effortful control and shyness at all time points and higher frustration at all but T1. None of the PRS were significantly related to affiliation. See Figure S5 for correlations between temperament outcomes at each time point and the adulthood psychopathology outcomes.

### Group-based temperament multi-trajectory models

**Figure 2.**
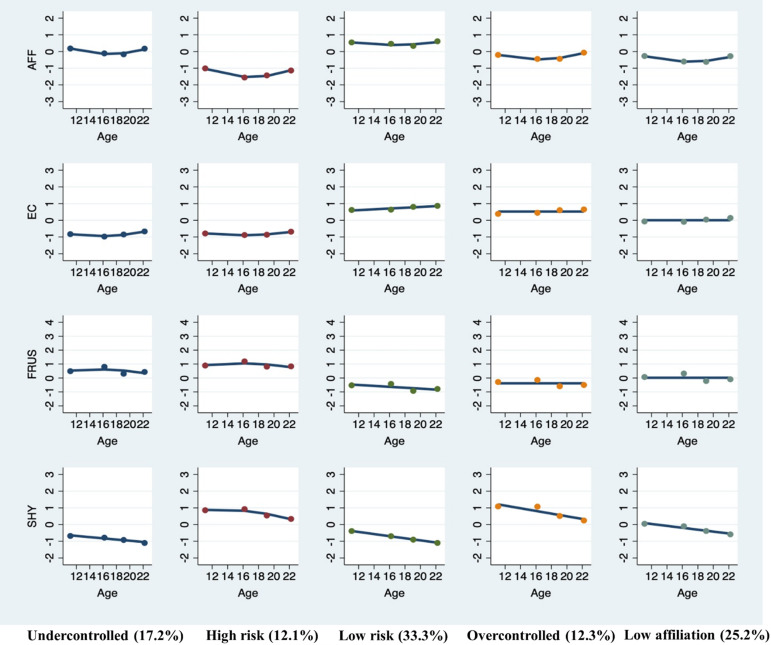
Temperament multi-trajectory groups. AFF = affiliation; EC = effortful control; FRUS = frustration; SHY = shyness. *X*-axis represents age (10 – 23 years old). *Y*-axis represents factor scores of temperament traits that exhibited partial strict invariance over all measurement occasions.

Analyses resulted in a choice of the 5- or 6-group model (Table 2). BIC was highest for the 5- and 6-group models. Model adequacy statistics for the 5- and 6-group solutions were similar, though the 5-group solution had higher entropy, suggesting greater accuracy in group assignments. In our evaluation of substantive interest of the models, we found that both solutions revealed overlapping low- and high-risk groups, but the low-risk group in the 5-group solution were split into two groups in the 6-group solution. This, combined with higher entropy, resulted in our choice of the 5-group solution (see Figure 2 and Table S4 for all parameter estimates).

**Table 2.** Model fit and adequacy statistics for estimated group-based multi-trajectory models

	Model fit		Model adequacy			
# Groups	Bayesian Information Criterion	Δ Bayesian Information Criterion	Range of Average posterior probability of group membership	Range of Odds of correct classification	SD of group membership probability	Entropy
1	−41,265.44	--				--
2	−37,961.02	3304.42				0.869
3	−36,685.45	1275.57				0.879
4	−35,735.20	950.25				0.879
5	−35,323.38	411.82	0.87 – 0.95	19.95 – 119.66	0.03	0.873
6	−34,869.86	453.52	0.89 – 0.94	27.82 – 197.86	0.04	0.857

To statistically test differences in temperament among each group, we conducted analyses of variance (ANOVAs) with post-hoc Tukey tests on the average item-level scores from each temperament dimension and timepoint. ANOVAs were all significant across every timepoint, suggesting that these trajectory groups are meaningfully different on all temperament dimensions (Table S5). Results of pairwise comparisons of temperament across trajectory groups were similar across T1–T4 (Figure S4 for visual depiction). Thus, results from T1 are shown for simplicity (Table S6). Most pairwise comparisons of temperament dimensions across trajectory groups were statistically significant. Based on comparisons and visualizations, there appears to be a *high-risk* group (*n* = 179) that is unique in showing the highest risk on all temperament traits (i.e., high frustration and shyness, low effortful control and affiliation). Next, there is a group that shows, relative to the *high-risk* group, similarly low effortful control and, relative to all groups, the second-highest frustration and affiliation and the lowest shyness (i.e., *undercontrolled* and exuberant group; *n* = 229). The next group is the inverse of the *undercontrolled* group, showing the second *lowest* frustration and second *highest* effortful control, while showing the highest shyness and average affiliation (i.e., *overcontrolled* and inhibited group; *n* = 167). Another group showed average levels of frustration, effortful control, and shyness, but the second lowest affiliation relative to all groups (*low affiliation*; *n* = 354). Finally, a *low-risk* group showed the most adaptive levels of temperament overall (*n* = 491).

### Predicting membership in temperament multi-trajectory groups

Tables 3 and S7 display results in which PRS and covariates predicted temperament multi-trajectory groups relative to the *low*- and *high-risk* groups. Figure S6 shows means of the PRS by temperament trajectory groups.

**Table 3.** Multinomial regressions predicting temperament multi-trajectory groups

		Low risk reference group		High risk reference group				
Trajectory	Predictor	Estimate	CI_95%_	OR		Estimate	CI_95%_	OR
Low risk (*n* = 491)	Intercept					2.08***	[1.73, 2.44]	8.03
	Depression PRS					−0.31**	[−0.50, −0.12]	0.73
	Externalizing PRS					0.04	[−0.16, 0.23]	1.04
	Sex					−1.15***	[−1.53, −0.77]	0.32
	Parental depression					−0.76***	[−1.16, −0.35]	0.47
	Parental anxiety					0.07	[−0.41, 0.55]	1.07
	Parental substance use					−0.48	[−1.21, 0.25]	0.62
	Parental antisocial					−0.13	[−0.84, 0.58]	0.88
	SES					0.40***	[0.21, 0.59]	1.49
Overcontrolled (*n* = 167)	Intercept	−1.13***	[−1.41, −0.85]	0.32		0.95***	[0.55, 1.36]	2.59
	Depression PRS	0.07	[−0.12, 0.26]	1.07		−0.24*	[−0.47, −0.01]	0.79
	Externalizing PRS	−0.29**	[−0.48, −0.10]	0.75		−0.25*	[−0.49, −0.01]	0.78
	Sex	−0.20	[−0.57, 0.17]	0.82		−1.35***	[−1.81, −0.89]	0.26
	Parental depression	0.39	[−0.02, 0.80]	1.48		−0.37	[−0.86, 0.12]	0.69
	Parental anxiety	−0.47	[−0.98, 0.04]	0.62		−0.40	[−1.00, 0.21]	0.67
	Parental substance use	0.25	[−0.58, 1.08]	1.29		−0.22	[−1.12, 0.67]	0.80
	Parental antisocial	0.26	[−0.51, 1.02]	1.29		0.13	[−0.73, 1.00]	1.14
	SES	−0.11	[−0.30, 0.08]	0.90		0.29*	[0.06, 0.52]	1.34
Low affiliation (*n* = 354)	Intercept	−0.73***	[−0.97, −0.50]	0.48		1.35***	[0.98, 1.72]	3.86
	Depression PRS	0.08	[−0.07, 0.23]	1.08		−0.23*	[−0.43, −0.03]	0.80
	Externalizing PRS	−0.07	[−0.21, 0.08]	0.94		−0.03	[−0.23, 0.17]	0.97
	Sex	0.73***	[0.44, 1.01]	2.07		−0.42*	[−0.82, −0.03]	0.65
	Parental depression	0.10	[−0.23, 0.43]	1.11		−0.65**	[−1.07, −0.24]	0.52
	Parental anxiety	−0.14	[−0.52, 0.25]	0.87		−0.07	[−0.56, 0.43]	0.94
	Parental substance use	0.86**	[0.25, 1.47]	2.36		0.38	[−0.30, 1.06]	1.47
	Parental antisocial	−0.21	[−0.83, 0.40]	0.81		−0.34	[−1.06, 0.38]	0.71
	SES	−0.22**	[−0.37, −0.08]	0.80		0.18	[−0.02, 0.37]	1.19
Undercontrolled (*n* = 229)	Intercept	−1.75***	[−2.06, −1.44]	0.17		0.33	[−0.08, 0.75]	1.40
	Depression PRS	0.17^	[0.00, 0.34]	1.18		−0.14	[−0.36, 0.07]	0.87
	Externalizing PRS	0.23**	[0.06, 0.41]	1.26		0.27*	[0.05, 0.49]	1.31
	Sex	1.05***	[0.72, 1.39]	2.87		−0.10	[−0.53, 0.33]	0.91
	Parental depression	0.87***	[0.50, 1.23]	2.38		0.11	[−0.33, 0.55]	1.12
	Parental anxiety	0.02	[−0.40, 0.44]	1.02		0.09	[−0.42, 0.61]	1.10
	Parental substance use	0.05	[−0.66, 0.75]	1.05		−0.43	[−1.18, 0.33]	0.65
	Parental antisocial	0.35	[−0.28, 0.97]	1.42		0.22	[−0.50, 0.95]	1.25
	SES	−0.07	[−0.24, 0.11]	0.94		0.34**	[0.12, 0.55]	1.40
High risk (*n* = 171)	Intercept	−2.08***	[−2.44, −1.73]	0.12				
	Depression PRS	0.31**	[0.12, 0.50]	1.36				
	Externalizing PRS	−0.04	[−0.23, 0.16]	0.96				
	Sex	1.15***	[0.77, 1.53]	3.17				
	Parental depression	0.76***	[0.35, 1.16]	2.13				
	Parental anxiety	−0.07	[−0.55, 0.41]	0.93				
	Parental substance use	0.48	[−0.25, 1.21]	1.61				
	Parental antisocial	0.13	[−0.58, 0.84]	1.14				
	SES	−0.40***	[−0.59, −0.21]	0.67				

*Note*. ^*p* < .06, *p < .05, **p < .01, ***p < .001. Sex: 0 = female; 1 = male; Parent psychopathology: 1 = present in father and/or mother; 0 = not present in either; OR = Odds Ratio; CI = 95% Confidence Interval. Models include controls for 10 ancestry components. See Table S7 for model estimates of ancestry components.

#### Prediction by PRS

**Figure 3.**
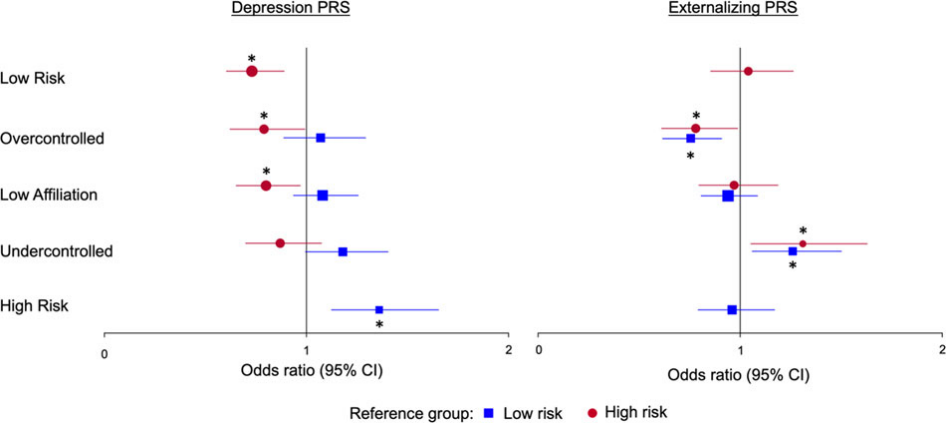
Forest plots of the multinomial regressions predicting temperament multi-trajectory groups. *Note*. This figure shows the odds ratios (dots and squares) and 95% CIs (visualized by the lines crossing the dots and squares) of the multinomial regressions predicting membership in the temperament multi-trajectory groups. The left plot visualizes the estimates for the depression PRS predicting group membership and the right plot shows the externalizing PRS predicting group membership. The colors represent the different models that were estimated, i.e., one where the *low*-*risk* group was the reference group (blue) and another where the *high-risk* group was the reference group (red). Significant odds ratios (*p* < .05) are identified with asterisks either below (for the model with the *low-risk* reference group in blue) or above (for the model with the *high-risk* reference group in red) the visual estimates.

See Figure 3. Levels of the externalizing PRS were higher in the *undercontrolled* and were lower in the *overcontrolled* groups, each relative to both *low*- and *high-risk* groups. Levels of the depression PRS were higher in the *high-risk* relative to the *low-risk*, *low-affiliation,* and *overcontrolled* groups.

#### Prediction by covariates

Males were more likely to belong to the *low affiliation*, *undercontrolled*, and *high-risk* relative to the *low-risk* group. Parental depression was overrepresented in the *undercontrolled* and *high-risk* relative to the *low-risk* group. Parental substance use was overrepresented in the *low affiliation* relative to the *low-risk* group. SES was lower in the *low-affiliation* and *high-risk* relative to the *low-risk* group. Analyzing models with covariates alone showed the same results.

### Predicting internalizing and externalizing outcomes

*Low*- and *high-risk* groups were examined as the reference group. See Figure S7 for a display of the means of all outcomes by temperament trajectory group.

#### Prediction of internalizing symptoms

**Figure 4.**
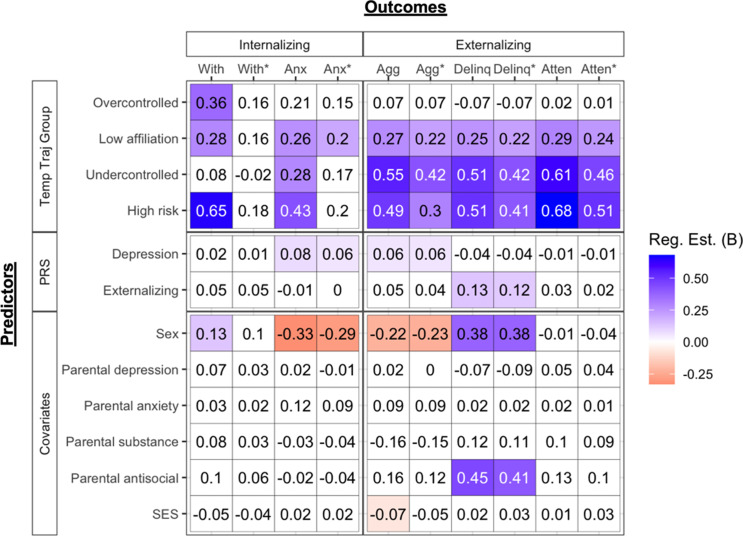
Heatmap of regression results predicting adulthood psychopathology outcomes from temperament multi-trajectory groups and PRS (Reference group = *low-risk*). Shaded cells represent regression estimates whose *p* < .05 (or *q* < 0.05 for primary variables). Non-significant estimates are in white. Estimates of 0 are non-zero and round to 0 at two decimal points. Abbreviations: “With/dep” = Withdrawal/depression, “Anx/dep” = Anxiety/depression, “Agg” = Aggressive behavior, “Delinq = Delinquent behavior, “Atten = Attention problems. Abbreviations with an asterisk (*) represent models where corresponding psychopathology covariates where included (e.g., with* = regression model that includes parent report of child withdrawal/depression at the first to third waves of data collection).

**Figure 5.**
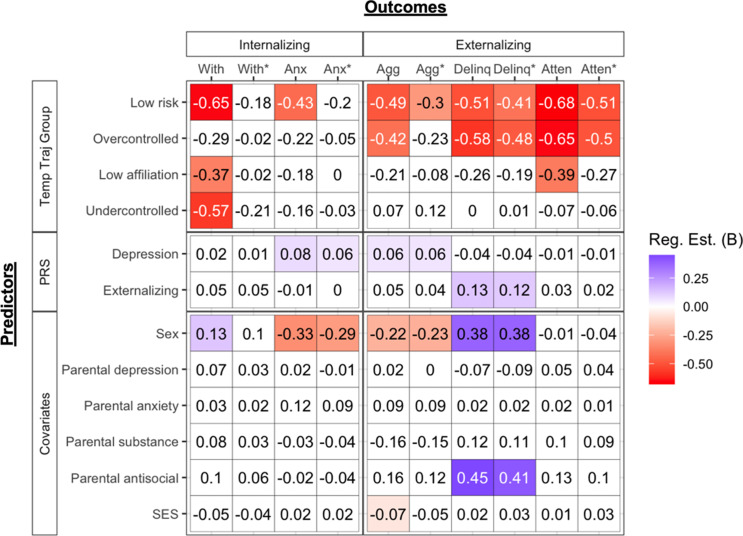
Heatmap of the regression results predicting alcohol, internalizing, and externalizing outcomes using temperament trajectory groups and PRS (Reference group = High risk). Shaded cells represent significant regression estimates at *p* < .05 (or *q* < 0.05 for primary variables). Non-significant estimates are in white. Estimates of 0 are non-zero and round to 0 at two decimal points. Abbreviations: “With/dep” = withdrawal/depression, “Anx/dep” = anxiety/depression, “Agg” = aggressive behavior, “Delinq” = delinquent behavior, “Atten” = attention problems. Abbreviations with an asterisk (*) represent models where corresponding psychopathology covariates where included (e.g., with*=regression model that includes parent report of child withdrawal/depression at the first to third waves of data collection).

See Figures 4 and 5 and Tables S8 and S10. Prior to controlling for earlier levels of psychopathology, the *high-risk* group showed significantly higher levels of withdrawn/depressed symptoms relative to all but the *overcontrolled* group, and the *overcontrolled* and *low-affiliation* groups showed higher levels of withdrawn/depressed symptoms than the *low-risk* group. Male sex was associated with higher withdrawal/depression. However, no temperament groups or covariates significantly predicted withdrawn/depressed symptoms after controlling for its earlier levels. The *high-risk, undercontrolled,* and *low-affiliation* groups showed higher levels of anxious/depressed symptoms relative to the *low-risk* group, but only the comparison with the *low-affiliation* group survived control for earlier levels of anxiety/depression. Higher levels of the depression PRS and female sex were associated with anxious/depressed symptoms across all models.

#### Prediction of externalizing symptoms

See Figures 4 and 5 and Tables S9 and S11. Prior to controlling for earlier psychopathology, the *high-risk*, *undercontrolled, and low-affiliation* groups showed higher levels of delinquency, aggression, and attentional problems relative to the *low-risk* group. All effects remained after controlling for earlier levels of psychopathology. Moreover, *the high-risk* group was higher on all three externalizing problems relative to the *overcontrolled* group and higher on attentional problems relative to the *low affiliation* group. After controlling for earlier psychopathology, the *high-risk* group no longer showed higher levels of aggression than the *overcontrolled* group nor higher levels of attentional problems relative to the *low affiliation* group. All externalizing outcomes had quite different patterns of associations with covariates. Higher levels of aggression were predicted by higher levels of the depression PRS, female sex, and lower SES across most models. Higher levels of delinquency were predicted by higher levels of the externalizing PRS, male sex, and parental antisocial behavior. Attentional problems showed no associations with covariates.

## Discussion

This was the first study to our knowledge to characterize how temperament dimensions of effortful control, frustration, affiliation, and shyness formed multi-trajectory groups from adolescence to early adulthood. We also examined correlates of these multi-trajectory groups, including PRS and future adulthood psychopathology. Using group-based multi-trajectory modeling, we found five temperament multi-trajectory groups whose patterns inform our understanding of temperament co-development. We also found meaningful differentiation of temperament trajectory groups by PRS and on later psychopathology, at times in ways that weren’t observed with individual temperament traits alone.

### Temperament multi-trajectory groups

The five groups found in this study were characterized by temperament trajectories we defined as *high-risk*, *undercontrolled*, *low-risk*, *overcontrolled*, and *low affiliation*. Temperament trajectory groups showed wide variation on not only levels, but slopes, of temperament trajectories. The *high-risk* group was the only one characterized by dips in maturation (i.e., quadratic slope) across all traits in adolescence. Relative to the other groups, the *high-risk* group generally showed the lowest affiliation, among the lowest effortful control, and the highest frustration and shyness; moreover, they were more likely to be male, to have parents with depression, and lower SES relative to the *low-risk* group. The *undercontrolled* group was similar to the *high-risk* group in levels and slopes of effortful control and frustration (i.e., dips in maturation) but compared to all other groups showed among the highest levels of affiliation and the lowest levels of shyness that showed no disruptions in maturation over time. Those in this group were more likely to be male and to have parental depression relative to the *low-risk* group.

Interestingly, the form of trait development in the *low-risk* group differed from the former two in that *maturation* was observed for all traits except affiliation (i.e., linear slopes in the direction of better temperament functioning). Relative to other groups, the *low-risk* group showed the highest effortful control and affiliation, lowest frustration, and relatively low shyness. Although the *overcontrolled* group showed slightly more risk on effortful control and frustration relative to the *low-risk* group, they notably did not show maturation but *stability* in these traits over time (i.e., flat, or no, development). Thus, despite somewhat adaptive functioning in these traits, individuals in the *overcontrolled* group lacked maturation that might be expected during the transition to adulthood. Those in the *overcontrolled* group also showed high but declining levels of shyness similar in levels to the *high-risk* group and overall moderate levels of affiliation; they were similar to the *low-risk* group on levels of covariates. Finally, the *low affiliation* group similarly showed flat development of effortful control and frustration over time, with levels similar to the *overcontrolled* group. In this group, affiliation was lower than all except the *high-risk* group and shyness was moderate and declining. Despite similarities to the *overcontrolled* group, the effect of covariates was quite different, with males, those with parental substance use, and lower SES more likely to be in the *low affiliation* than *low-risk* group.

The characterization of temperament multi-trajectory groups provides greater context against which to understand the mixed literature on temperament development from adolescence to adulthood. That is, youth may experience either maturation, disruption, *or* little change in the development of effortful control, frustration, affiliation, and shyness from adolescence to adulthood. Finding groups with relatively flat development of temperament traits over time (i.e., *overcontrolled* and *low affiliation*) also suggests that *lack of* maturation may be important to consider in understanding risk for negative outcomes. Moreover, the number of temperament traits that showed disruptions (i.e., quadratic slopes) in adolescence were generally higher among groups showing poorer temperament functioning (i.e., *high-risk* and *undercontrolled*), the number of traits that showed maturation was highest in the *low-risk* group, and the number of traits that showed no change was more common in groups that could be considered intermediate in risk (i.e., *overcontrolled* and *low affiliation*). Mixed results in the literature on the development of temperament may reflect differences in sample composition that skew findings more heavily towards one pattern or another.

One exception was that affiliation uniformly showed disruption in adolescence across all groups. This may reflect the fact that adolescence is a period of autonomy seeking from parental figures, leading parents to report that their adolescents became less warm and friendly during this time (McElhaney & Allen, 2001). Another interesting observation was that effortful control and frustration trajectories were mirror images with respect to their overall levels and rates of change within each group (see Figure 2). Although prior research has shown that these traits are moderately to highly correlated (Snyder et al., 2015), our results further highlight the intimate relationship between effortful control and frustration across adolescence and early adulthood and call for further research to understand their shared biological and environmental causes.

### Polygenic risk scores predicting temperament multi-trajectory groups

The most striking finding along these lines was that the *undercontrolled* group showed elevated levels of the externalizing PRS, and the *overcontrolled* group showed lower levels of the externalizing PRS, relative to *both low*- and *high-risk* groups (see Figure S6). Zero-order correlations suggested that this effect was not simply driven by one or two temperament trajectories. Indeed, the externalizing PRS was most strongly correlated with lower effortful control at all time points and, to a lesser degree, with higher frustration and lower shyness (Table S3). In fact, it was the *high*- and *low-risk* groups, not *undercontrolled* or *overcontrolled*, that showed the most extreme levels of effortful control and frustration.

It is interesting that the externalizing PRS showed more specific prediction of temperament multi-trajectory groups, especially given its pleiotropic effects on individual temperament traits. Temperament groups represent the “whole person” with respect to temperament traits, which may be helpful in unpacking the meaning of widely used PRS. Results suggest that those with higher externalizing PRS may be at risk for a unique collection of temperamental traits reflecting high levels of dysregulation (i.e., low effortful control, high frustration) coupled with high exuberance (i.e., affiliation and low shyness; Degnan et al., 2011).

We also found that higher levels of the depression PRS predicted membership in the *high-risk* relative to *low-risk, overcontrolled,* and *low-affiliation* groups (but not relative to the *undercontrolled* group). The observed associations with the depression PRS may be driven by effortful control and frustration. Indeed, the *high-risk* and *undercontrolled* groups are similar in their high frustration and low effortful control, and zero-order correlations showed that the depression PRS significantly correlated with only effortful control and frustration across all assessments. Thus, temperament multi-trajectory groups do not appear to provide greater specificity in associations with the depression PRS relative to temperament traits alone. Note that, although the depression and externalizing PRS were correlated, each of their effects on group membership were independent of one another, as both PRS were entered simultaneously into regression models. Finally, it is interesting to note that parental depression was elevated in the same multi-trajectory groups in which the depression PRS was highest: *undercontrolled* and *high-risk*. The fact that both parental depression and the depression PRS predicted temperament groups characterized by low effortful control and high frustration further suggests that, far from being specific to depression, parental depression and depression PRS index a general propensity towards dysregulation.

### Temperament multi-trajectory groups predicting adulthood psychopathology

Prior to and after controlling for earlier psychopathology, the *high-risk*, *undercontrolled,* and *low-affiliation* groups generally showed the highest levels (in that order) of delinquency, aggression, and attentional problems relative to *low-risk* and *overcontrolled* groups (also see Figure S7). Moreover, the *high-risk* and *undercontrolled* groups did not differ on these outcomes, suggesting they carry the highest risk. The *high-risk, undercontrolled,* and *low-affiliation* groups also showed heightened risk for anxious/depressed symptoms relative to the *low-risk* group, with only the effect from *low affiliation* surviving control for earlier anxious depression. These groups (*high-risk, undercontrolled, low affiliation*) showed the first, second, and third highest risk on frustration and effortful control, respectively. Thus, results are likely driven by these two traits and are consistent with prior studies showing that the interaction between effortful control and negative affectivity is important in the development of externalizing and internalizing problems (Gartstein et al., 2012; Oldehinkel et al., 2007).

In light of their similar levels of future externalizing problems, it is noteworthy that the *high-risk* and *undercontrolled* groups were predicted by different PRS. This implies that there may be two genetic pathways to externalizing problems: one emanating from the externalizing PRS that operates through risk for undercontrolled and exuberant temperament traits, and the second from the depression PRS that operates through risk for dysregulation and poorer socially oriented traits (e.g., shyness, low affiliation).

We also found that the *high-risk* group was uniquely elevated on withdrawn depression relative to all but the *overcontrolled* group. That the *high-risk* group showed heightened risk echoes a replicated finding that the combination of high neuroticism, low extraversion, and low conscientiousness presented high risk for depression (Vasey et al., 2013, 2014). Additionally, the *overcontrolled* and *low-affiliation* groups showed higher withdrawal/depression relative to the *low-risk* group and the *undercontrolled* and *low-risk* groups did not differ (also see Figure S7). Interestingly, the *overcontrolled* and *low affiliation* groups were not particularly heightened on effortful control and frustration. Moreover, the *high-risk*, *overcontrolled*, and *low-affiliation* groups showed the highest levels of shyness and lowest levels of affiliation, generally in that order. This raises the possibility that poorer functioning on these socially oriented traits (i.e., shyness, low affiliation) may be more prominent over other traits (i.e., effortful control, frustration) in the development of withdrawal/depression for some individuals. Withdrawn depression was also one of the few ways in which the *high-risk* and *undercontrolled* groups differed, suggesting that greater affiliation and lower shyness (unique characteristics of *undercontrolled* group) can buffer individuals with low regulatory abilities from experiencing an even broader range of negative mental health outcomes. Because none of these effects survived controlling for earlier withdrawn depression, these temperament groups may not be useful above and beyond concurrent withdrawn depression for forecasting future risk.

Several limitations and strengths should be considered in the interpretation of these findings. The sample consisted of White individuals from the Netherlands, limiting generalizability. Constraining the sample to those of European ancestry was needed to reduce concerns of bias from population stratification but is nonetheless not a generalizable approach. Moreover, this dataset unfortunately did not measure temperament between the ages of 11 – 16 when changes in temperament (particularly disruptions) often occur. Nonetheless, our study was still able to detect disruptions that occur in temperament development during this time, as evidenced by significant quadratic slopes. Perhaps if we had more data, these disruptions in temperament development would have been more robust. Finally, although research showed different relative contributions from genetic and environmental influences on temperament profiles in youth (Murillo et al., 2024), our study was unable to disentangle genetic and environmental influences. This would be an important future direction, especially given the potential importance of stability versus change in understanding genetic and environmental influences on temperament (Ganiban et al., 2008). We also did not adjust for clustering by school membership, as the available data had considerable missingness whose inclusion would have resulted in the loss of almost 30% of cases. Moreover, the academic trajectories of youth in the Netherlands over the five waves studied were likely to shift considerably over time, suggesting that school membership at any one wave may not have a substantial impact on the study variables. Strengths include the richly phenotyped longitudinal dataset, rigorous measurement, integration of multimodal data, and novel examination of co-occurrence among trajectories of temperament during the critical period from adolescence to early adulthood.

## Conclusion

Results shed light on the developmental patterning of temperament traits from adolescence to adulthood, demonstrating that there may exist systematic combinations of temperament trajectories that differ in both levels and form over adolescence and young adulthood. The observed temperament multi-trajectory groups could also have importance for further etiologic inquiry and prognosis. For example, the genetic backgrounds underlying the *high-risk* and *undercontrolled* groups could be examined in future studies to better characterize two distinct genetic pathways to externalizing problems. Moreover, individuals who consistently show high risk on all temperament traits (i.e., low effortful control and affiliation, high shyness and frustration) could be targeted for transdiagnostic mental health prevention programs given high risk for many forms of psychopathology, whereas individuals showing profiles similar to the *undercontrolled group* could be provided with early prevention for externalizing problems only.

## Supporting information

10.1017/S0954579425100680.sm001 Wang et al. supplementary material Wang et al. supplementary material

## Data Availability

TRAILS data are not open source but accessible for researchers outside the TRAILS consortium by submitting a publication proposal (www.trails.nl/en/home). The code for this study can be provided upon reasonable request to the corresponding author.

## Supplementary material

The supplementary material for this article can be found at https://doi.org/10.1017/S0954579425100680.
