# Supplementary material for: Temperament multi-trajectory groups across adolescence: Associations with adulthood psychopathology and polygenic scores in TRAILS
Source: Dev Psychopathol. 2025 Sep 17:1–15. Online ahead of print. doi: 10.1017/S0954579425100680 (PMC12752481; doi:10.1017/S0954579425100680)
Supplement: Wang et al. supplementary material [file S0954579425100680sup001.docx]

Table S1. Appendix of temperament items

| Construct | Variable Name | Item |
| --- | --- | --- |
| Effortful Control | EC1 | It takes a lot of effort for my child to get things done on time |
| Effortful Control | EC2 | My child finds it easy to concentrate well on a task |
| Effortful Control | EC3 | If my child has to do a difficult task, he / she usually starts right away |
| Effortful Control | EC4 | My child usually puts off things to do until the last minute |
| Effortful Control | EC5 | My child is already going to do something else before what he / she is doing is finished |
| Effortful Control | EC6 | If my child has a plan, he / she usually manages to keep it up until he / she has reached the goal |
| Effortful Control | EC7 | My child pays close attention when someone explains how to do something |
| Affiliation | AFF1 | My child likes to take care of others |
| Affiliation | AFF2 | My child likes to be able to talk to someone about everything he / she thinks |
| Affiliation | AFF3 | My child finds it important to have a good relationship with others |
| Affiliation | AFF4 | My child is a warm and friendly person |
| Frustation | FRUS1 | My child is annoyed by little things that other children do |
| Frustation | FRUS2 | My child gets very irritated when someone criticizes him / her |
| Frustation | FRUS3 | My child gets irritated when he / she has to stop doing something he / she likes |
| Frustation | FRUS4 | My child hates it when others disagree with him / her |
| Shyness | SHY1 | My child knows how to say something in company, even with strangers |
| Shyness | SHY2 | My child is shy when he / she meets new people |

Table S2. *Summary of invariance testing across time and sex for each temperament construct*

| **Assessment Period** | | | | | | | | | |
| --- | --- | --- | --- | --- | --- | --- | --- | --- | --- |
| Model | RMSEA | | RMSEA 90% CI | ΔRMSEA | CFI | ΔCFI | SRMR | BIC | ΔBIC |
| **Affiliation** | | | | | | | | | |
| Configural | | 0.026 | [0.021, 0.031] | - | 0.988 | - | 0.022 | 69498.12 | - |
| Metric | | 0.025 | [0.021, 0.030] | -0.001 | 0.987 | -0.001 | 0.026 | 69471.93 | -26.19 |
| Scalar | | 0.038 | [0.034, 0.042] | 0.013 | 0.968 | -0.019 | 0.035 | 69620.69 | 148.75 |
| **Effortful control** | | | | | | | | | |
| Configural | | 0.046 | [0.043, 0.047] | - | 0.941 | - | 0.036 | 128474.33 | - |
| Metric | | 0.047 | [0.045, 0.049] | 0.001 | 0.936 | -0.005 | 0.045 | 128534.92 | 60.59 |
| Scalar | | 0.054 | [0.050, 0.054] | 0.007 | 0.910 | -0.026 | 0.049 | 129107.89 | 572.97 |
| Partial scalar | | 0.041 | [0.049, 0.053] | -0.006 | 0.894 | -0.016 | 0.045 | 128860.01 | 325.09 |
| **Frustration** | |  |  |  |  |  |  |  |  |
| Configural | | 0.022 | [0.017, 0.027] | - | 0.992 | - | 0.023 | 74137.31 | - |
| Metric | | 0.025 | [0.020, 0.030] | 0.003 | 0.988 | -0.004 | 0.033 | 74143.11 | 5.797 |
| Scalar | | 0.061 | [0.057, 0.065] | 0.036 | 0.923 | -0.065 | 0.044 | 74733.58 | 590.47 |
| Partial scalar | | 0.049 | [0.043, 0.051] | 0.024 | 0.952 | -0.036 | 0.035 | 74467.87 | 324.76 |
| **Sex** | | | | | | | | | |
| Model | | RMSEA | RMSEA 90% CI | ΔRMSEA | CFI | ΔCFI | SRMR |  |  |
| **Affiliation** | |  |  |  |  |  |  |  |  |
| Configural | | 0.093 | [0.088, 0.097] | - | 0.964 | - | 0.075 |  |  |
| Metric | | 0.091 | [0.087, 0.096] | -0.002 | 0.963 | -0.001 | 0.076 |  |  |
| Scalar | | 0.090 | [0.086, 0.094] | -0.001 | 0.957 | -0.006 | 0.076 |  |  |
| **Effortful Control** | | | | | | | | | |
| Configural | | 0.073 | [0.070, 0.075] | - | 0.983 | - | 0.059 |  |  |
| Metric | | 0.072 | [0.070, 0.075] | -0.001 | 0.982 | -0.001 | 0.060 |  |  |
| Scalar | | 0.069 | [0.066, 0.071] | -0.003 | 0.982 | 0.000 | 0.060 |  |  |
| **Frustration** | |  |  |  |  |  |  |  |  |
| Configural | | 0.067 | [0.062, 0.071] | - | 0.982 | - | 0.056 |  |  |
| Metric | | 0.067 | [0.063, 0.072] | 0.000 | 0.981 | -0.001 | 0.058 |  |  |
| Scalar | | 0.065 | [0.061, 0.069] | -0.002 | 0.979 | -0.002 | 0.057 |  |  |
| **Shyness** | |  |  |  |  |  |  |  |  |
| Configural/ Metric | | 0.056 | [0.045, 0.067] | - | 0.996 | - | 0.039 |  |  |
| Scalar | | 0.046 | [0.036, 0.055] | -0.010 | 0.996 | 0.000 | 0.037 |  |  |

*Note.* Since the shyness dimension consisted of two items, the configural and metric models are equivalent.

Table S3. Zero-order correlations between Polygenic Risk Scores (PRS) and temperament at each assessment.

|  | Depression PRS | Externalizing PRS |  |  |  |
| --- | --- | --- | --- | --- | --- |
| AFF 1 | 0.01 | 0.03 |  |  |  |
| AFF 2 | -0.02 | 0.00 |  |  |  |
| AFF 3 | -0.04 | -0.01 |  |  |  |
| AFF 4 | -0.01 | 0.01 |  | Significance | |
| EC 1 | -0.08 | -0.12 |  |  | Non-sig (*p*>=.05) |
| EC 2 | -0.10 | -0.15 |  |  | *p*<.001 |
| EC 3 | -0.09 | -0.13 |  |  | *p*<.01 |
| EC 4 | -0.11 | -0.14 |  |  | *p*<.05 |
| FRUS 1 | 0.09 | 0.05 |  |  |  |
| FRUS 2 | 0.12 | 0.08 |  |  |  |
| FRUS 3 | 0.12 | 0.08 |  |  |  |
| FRUS 4 | 0.14 | 0.09 |  |  |  |
| SHY 1 | 0.00 | -0.14 |  |  |  |
| SHY 2 | 0.03 | -0.09 |  |  |  |
| SHY 3 | 0.05 | -0.06 |  |  |  |
| SHY 4 | 0.04 | -0.06 |  |  |  |

Note. EC: Effortful Control. AFF: Affiliation. FRUS: Frustration. SHY: Shyness.

Table S4. *Group-based trajectory model estimates (5-group solution)*

| **Affiliation (AFF)** | | | | | |
| --- | --- | --- | --- | --- | --- |
| Group | Parameter (Slope) | Estimate | SE | T for H0 | Prob > \|T\| |
| 1 | Intercept | 2.68 | 0.36 | 7.35 | 0.00 |
|  | Linear | -0.34 | 0.05 | -7.27 | 0.00 |
|  | Quadratic | 0.01 | 0.00 | 7.17 | 0.00 |
| 2 | Intercept | 2.66 | 0.44 | 6.04 | 0.00 |
|  | Linear | -0.50 | 0.06 | -8.84 | 0.00 |
|  | Quadratic | 0.01 | 0.00 | 8.63 | 0.00 |
| 3 | Intercept | 1.85 | 2.26 | 7.25 | 0.00 |
|  | Linear | -0.28 | 0.03 | -5.47 | 0.00 |
|  | Quadratic | 0.01 | 0.00 | 5.51 | 0.00 |
| 4 | Intercept | 2.29 | 0.43 | 5.31 | 0.00 |
|  | Linear | -0.34 | 0.05 | -6.25 | 0.00 |
|  | Quadratic | 0.01 | 0.00 | 6.41 | 0.00 |
| 5 | Intercept | 2.24 | 0.30 | 7.41 | 0.00 |
|  | Linear | -0.34 | 0.04 | -8.69 | 0.00 |
|  | Quadratic | 0.01 | 0.00 | 8.55 | 0.00 |
|  | Sigma | 0.73 | 0.01 | 91.59 | 0.00 |
| **Effortful Control (EC)** | | | | | |
| Group | Parameter | Estimate | SE | T for H0 | Prob > \|T\| |
| 1 | Intercept | 0.51 | 0.34 | 1.49 | 0.13 |
|  | Linear | -0.19 | 0.04 | -4.36 | 0.00 |
|  | Quadratic | 0.01 | 0.00 | 4.69 | 0.00 |
| 2 | Intercept | 0.31 | 0.42 | 0.75 | 0.45 |
|  | Linear | -0.15 | 0.05 | -2.89 | 0.00 |
|  | Quadratic | 0.00 | 0.00 | 3.02 | 0.00 |
| 3 | Intercept | 0.33 | 0.06 | 5.57 | 0.00 |
|  | Linear | 0.02 | 0.00 | 7.14 | 0.00 |
| 4 | Intercept | 0.53 | 0.30 | 17.62 | 0.00 |
| 5 | Intercept | 0.01 | 0.03 | 0.27 | 0.78 |
|  | Sigma | 0.68 | 0.01 | 98.32 | 0.00 |
| **Frustration (FRUS)** | | | | | |
| Group | Parameter | Estimate | SE | T for H0 | Prob > \|T\| |
| 1 | Intercept | -0.60 | 0.39 | -1.54 | 0.12 |
|  | Linear | 0.16 | 0.05 | 3.25 | 0.00 |
|  | Quadratic | -0.01 | 0.00 | -3.58 | 0.00 |
| 2 | Intercept | -0.43 | 0.47 | -0.91 | 0.36 |
|  | Linear | 0.19 | 0.06 | 3.18 | 0.00 |
|  | Quadratic | -0.01 | 0.00 | -3.39 | 0.00 |
| 3 | Intercept | -0.11 | 0.07 | -1.58 | 0.11 |
|  | Linear | -0.03 | 0.00 | -8.77 | 0.00 |
| 4 | Intercept | -0.38 | 0.03 | -11.22 | 0.00 |
| 5 | Intercept | 0.02 | 0.03 | 0.56 | 0.58 |
|  | Sigma | 0.78 | 0.01 | 103.13 | 0.00 |
| **Shyness (SHY)** | | | | | |
| Group | Parameter | Estimate | SE | T for H0 | Prob > \|T\| |
| 1 | Intercept | -0.24 | 0.07 | -3.28 | 0.00 |
|  | Linear | -0.04 | 0.00 | -9.17 | 0.00 |
| 2 | Intercept | -0.18 | 0.35 | -0.51 | 0.61 |
|  | Linear | 0.17 | 0.05 | 3.72 | 0.00 |
|  | Quadratic | -0.01 | 0.00 | -4.82 | 0.00 |
| 3 | Intercept | 0.33 | 0.05 | 6.40 | 0.00 |
|  | Linear | -0.06 | 0.00 | -22.82 | 0.00 |
| 4 | Intercept | 2.09 | 0.09 | 23.80 | 0.00 |
|  | Linear | -0.08 | 0.00 | -16.17 | 0.00 |
| 5 | Intercept | 0.72 | 0.06 | 11.14 | 0.00 |
|  | Linear | -0.06 | 0.00 | -16.25 | 0.00 |
|  | Sigma | 0.58 | 0.01 | 90.82 | 0.00 |

Table S5. *Results of the ANOVAs for differences in temperament between the trajectory groups*

| Temperament Dimension | Timepoint | F-value | *p* |
| --- | --- | --- | --- |
| Affiliation | 1 | 135.20 | < 0.001 |
| Affiliation | 3 | 197.40 | < 0.001 |
| Affiliation | 4 | 151.00 | < 0.001 |
| Affiliation | 5 | 142.60 | < 0.001 |
| Effortful Control | 1 | 167.90 | < 0.001 |
| Effortful Control | 3 | 266.80 | < 0.001 |
| Effortful Control | 4 | 309.10 | < 0.001 |
| Effortful Control | 5 | 240.60 | < 0.001 |
| Frustration | 1 | 121.20 | < 0.001 |
| Frustration | 3 | 149.30 | < 0.001 |
| Frustration | 4 | 161.00 | < 0.001 |
| Frustration | 5 | 160.90 | < 0.001 |
| Shyness | 1 | 174.00 | < 0.001 |
| Shyness | 3 | 302.10 | < 0.001 |
| Shyness | 4 | 204.40 | < 0.001 |
| Shyness | 5 | 196.40 | < 0.001 |

Table S6. *Results of the post-hoc Tukey tests for differences in temperament between the trajectory groups (T1 only)*

|  | |  | *p*-value of comparison (Group vs. _______) | | | | |
| --- | --- | --- | --- | --- | --- | --- | --- |
| Temperament dimension | Group | *Mean* | *SD* | High risk | Undercontrolled | Low affiliation | Overcontrolled |
| Affiliation | High risk | 3.25 | 0.66 |  |  |  |  |
|  | Undercontrolled | 4.07 | 0.52 | <.001 |  |  |  |
|  | Low affiliation | 3.76 | 0.53 | <.001 | <.001 |  |  |
|  | Overcontrolled | 3.67 | 0.59 | <.001 | <.001 | 0.403 |  |
|  | Low risk | 4.27 | 0.46 | <.001 | <.001 | <.001 | <.001 |
| Effortful control | High risk | 2.67 | 0.66 |  |  |  |  |
|  | Undercontrolled | 2.68 | 0.61 | <.001 |  |  |  |
|  | Low affiliation | 3.25 | 0.58 | <.001 | <.001 |  |  |
|  | Overcontrolled | 3.60 | 0.63 | <.001 | <.001 | <.001 |  |
|  | Low risk | 3.74 | 0.62 | <.001 | <.001 | <.001 | 0.092 |
| Frustration | High risk | 3.54 | 0.59 |  |  |  |  |
|  | Undercontrolled | 3.22 | 0.63 | <.001 |  |  |  |
|  | Low affiliation | 2.95 | 0.57 | <.001 | <.001 |  |  |
|  | Overcontrolled | 2.72 | 0.63 | <.001 | <.001 | <.001 |  |
|  | Low risk | 2.50 | 0.58 | <.001 | <.001 | <.001 | <.001 |
| Shyness | High risk | 3.14 | 0.92 |  |  |  |  |
|  | Undercontrolled | 1.77 | 0.66 | <.001 |  |  |  |
|  | Low affiliation | 2.48 | 0.69 | <.001 | <.001 |  |  |
|  | Overcontrolled | 3.45 | 0.79 | 0.002 | <.001 | <.001 |  |
|  | Low risk | 2.12 | 0.75 | <.001 | <.001 | <.001 | <.001 |

Table S7. *Multinomial regressions predicting temperament multi-trajectory groups*

|  |  | Low risk reference group | | | High risk reference group | | |
| --- | --- | --- | --- | --- | --- | --- | --- |
| Trajectory | Predictor | Estimate | CI_95%_ | OR | Estimate | CI_95%_ | OR |
| Low risk  (*n*=491) | Intercept |  |  |  | 2.08*** | [1.73, 2.44] | 8.03 |
|  | Depression PRS |  |  |  | -0.31** | [-0.50, -0.12] | 0.73 |
|  | Externalizing PRS |  |  |  | 0.04 | [-0.16, 0.23] | 1.04 |
|  | Sex |  |  |  | -1.15*** | [-1.53, -0.77] | 0.32 |
|  | Parental depression |  |  |  | -0.76*** | [-1.16, -0.35] | 0.47 |
|  | Parental anxiety |  |  |  | 0.07 | [-0.41, 0.55] | 1.07 |
|  | Parental substance use |  |  |  | -0.48 | [-1.21, 0.25] | 0.62 |
|  | Parental antisocial |  |  |  | -0.13 | [-0.84, 0.58] | 0.88 |
|  | SES |  |  |  | 0.40*** | [0.21, 0.59] | 1.49 |
|  | Ancestry PC1 |  |  |  | -0.20* | [-0.39, -0.01] | 0.82 |
|  | Ancestry PC2 |  |  |  | 0.16 | [-0.03, 0.36] | 1.18 |
|  | Ancestry PC3 |  |  |  | 0.24* | [0.04, 0.43] | 1.27 |
|  | Ancestry PC4 |  |  |  | 0.11 | [-0.11, 0.34] | 1.12 |
|  | Ancestry PC5 |  |  |  | 0.04 | [-0.17, 0.25] | 1.04 |
|  | Ancestry PC6 |  |  |  | -0.06 | [-0.27, 0.15] | 0.94 |
|  | Ancestry PC7 |  |  |  | -0.11 | [-0.34, 0.12] | 0.90 |
|  | Ancestry PC8 |  |  |  | 0.11 | [-0.09, 0.32] | 1.12 |
|  | Ancestry PC9 |  |  |  | -0.02 | [-0.23, 0.20] | 0.99 |
|  | Ancestry PC10 |  |  |  | 0.09 | [-0.12, 0.30] | 1.09 |
| Overcontrolled  (*n*=167) | Intercept | -1.13*** | [-1.41, -0.85] | 0.32 | 0.95*** | [0.55, 1.36] | 2.59 |
|  | Depression PRS | 0.07 | [-0.12, 0.26] | 1.07 | -0.24* | [-0.47, -0.01] | 0.79 |
|  | Externalizing PRS | -0.29** | [-0.48, -0.10] | 0.75 | -0.25* | [-0.49, -0.01] | 0.78 |
|  | Sex | -0.20 | [-0.57, 0.17] | 0.82 | -1.35*** | [-1.81, -0.89] | 0.26 |
|  | Parental depression | 0.39 | [-0.02, 0.80] | 1.48 | -0.37 | [-0.86, 0.12] | 0.69 |
|  | Parental anxiety | -0.47 | [-0.98, 0.04] | 0.62 | -0.40 | [-1.00, 0.21] | 0.67 |
|  | Parental substance use | 0.25 | [-0.58, 1.08] | 1.29 | -0.22 | [-1.12, 0.67] | 0.80 |
|  | Parental antisocial | 0.26 | [-0.51, 1.02] | 1.29 | 0.13 | [-0.73, 1.00] | 1.14 |
|  | SES | -0.11 | [-0.30, 0.08] | 0.90 | 0.29* | [0.06, 0.52] | 1.34 |
|  | Ancestry PC1 | 0.07 | [-0.12, 0.26] | 1.07 | -0.13 | [-0.36, 0.11] | 0.88 |
|  | Ancestry PC2 | -0.04 | [-0.23, 0.14] | 0.96 | 0.12 | [-0.11, 0.35] | 1.13 |
|  | Ancestry PC3 | -0.09 | [-0.28, 0.11] | 0.92 | 0.15 | [-0.09, 0.39] | 1.16 |
|  | Ancestry PC4 | -0.18 | [-0.40, 0.04] | 0.83 | -0.07 | [-0.34, 0.21] | 0.94 |
|  | Ancestry PC5 | -0.18 | [-0.39, 0.04] | 0.84 | -0.14 | [-0.40, 0.13] | 0.87 |
|  | Ancestry PC6 | -0.13 | [-0.33, 0.07] | 0.88 | -0.19 | [-0.45, 0.06] | 0.83 |
|  | Ancestry PC7 | 0.22 | [0.00, 0.43] | 1.24 | 0.11 | [-0.17, 0.38] | 1.11 |
|  | Ancestry PC8 | 0.11 | [-0.08, 0.30] | 1.11 | 0.22 | [-0.02, 0.46] | 1.25 |
|  | Ancestry PC9 | -0.04 | [-0.25, 0.17] | 0.96 | -0.05 | [-0.32, 0.21] | 0.95 |
|  | Ancestry PC10 | -0.09 | [-0.27, 0.10] | 0.92 | 0.00 | [-0.23, 0.24] | 1.00 |
| Low affiliation  (*n*=354) | Intercept | -0.73*** | [-0.97, -0.50] | 0.48 | 1.35*** | [0.98, 1.72] | 3.86 |
|  | Depression PRS | 0.08 | [-0.07, 0.23] | 1.08 | -0.23* | [-0.43, -0.03] | 0.80 |
|  | Externalizing PRS | -0.07 | [-0.21, 0.08] | 0.94 | -0.03 | [-0.23, 0.17] | 0.97 |
|  | Sex | 0.73*** | [0.44, 1.01] | 2.07 | -0.42* | [-0.82, -0.03] | 0.65 |
|  | Parental depression | 0.10 | [-0.23, 0.43] | 1.11 | -0.65** | [-1.07, -0.24] | 0.52 |
|  | Parental anxiety | -0.14 | [-0.52, 0.25] | 0.87 | -0.07 | [-0.56, 0.43] | 0.94 |
|  | Parental substance use | 0.86** | [0.25, 1.47] | 2.36 | 0.38 | [-0.30, 1.06] | 1.47 |
|  | Parental antisocial | -0.21 | [-0.83, 0.40] | 0.81 | -0.34 | [-1.06, 0.38] | 0.71 |
|  | SES | -0.22** | [-0.37, -0.08] | 0.80 | 0.18 | [-0.02, 0.37] | 1.19 |
|  | Ancestry PC1 | 0.11 | [-0.04, 0.26] | 1.12 | -0.09 | [-0.28, 0.11] | 0.92 |
|  | Ancestry PC2 | -0.08 | [-0.22, 0.07] | 0.93 | 0.09 | [-0.11, 0.28] | 1.09 |
|  | Ancestry PC3 | -0.12 | [-0.27, 0.04] | 0.89 | 0.12 | [-0.08, 0.32] | 1.12 |
|  | Ancestry PC4 | 0.06 | [-0.10, 0.23] | 1.06 | 0.18 | [-0.05, 0.40] | 1.19 |
|  | Ancestry PC5 | -0.12 | [-0.28, 0.04] | 0.89 | -0.08 | [-0.30, 0.13] | 0.92 |
|  | Ancestry PC6 | 0.13 | [-0.03, 0.28] | 1.13 | 0.07 | [-0.15, 0.28] | 1.07 |
|  | Ancestry PC7 | -0.01 | [-0.17, 0.16] | 0.99 | -0.12 | [-0.35, 0.12] | 0.89 |
|  | Ancestry PC8 | 0.00 | [-0.15, 0.15] | 1.00 | 0.12 | [-0.09, 0.33] | 1.12 |
|  | Ancestry PC9 | 0.05 | [-0.10, 0.21] | 1.05 | 0.04 | [-0.18, 0.26] | 1.04 |
|  | Ancestry PC10 | 0.01 | [-0.14, 0.16] | 1.01 | 0.10 | [-0.11, 0.30] | 1.10 |
| Undercontrolled  (*n*=229) | Intercept | -1.75*** | [-2.06, -1.44] | 0.17 | 0.33 | [-0.08, 0.75] | 1.40 |
|  | Depression PRS | 0.17^ | [0.00, 0.34] | 1.18 | -0.14 | [-0.36, 0.07] | 0.87 |
|  | Externalizing PRS | 0.23** | [0.06, 0.41] | 1.26 | 0.27* | [0.05, 0.49] | 1.31 |
|  | Sex | 1.05*** | [0.72, 1.39] | 2.87 | -0.10 | [-0.53, 0.33] | 0.91 |
|  | Parental depression | 0.87*** | [0.50, 1.23] | 2.38 | 0.11 | [-0.33, 0.55] | 1.12 |
|  | Parental anxiety | 0.02 | [-0.40, 0.44] | 1.02 | 0.09 | [-0.42, 0.61] | 1.10 |
|  | Parental substance use | 0.05 | [-0.66, 0.75] | 1.05 | -0.43 | [-1.18, 0.33] | 0.65 |
|  | Parental antisocial | 0.35 | [-0.28, 0.97] | 1.42 | 0.22 | [-0.50, 0.95] | 1.25 |
|  | SES | -0.07 | [-0.24, 0.11] | 0.94 | 0.34** | [0.12, 0.55] | 1.40 |
|  | Ancestry PC1 | 0.18* | [0.01, 0.35] | 1.20 | -0.02 | [-0.23, 0.19] | 0.98 |
|  | Ancestry PC2 | 0.03 | [-0.14, 0.20] | 1.03 | 0.19 | [-0.03, 0.41] | 1.21 |
|  | Ancestry PC3 | -0.04 | [-0.22, 0.15] | 0.97 | 0.20 | [-0.02, 0.43] | 1.22 |
|  | Ancestry PC4 | 0.04 | [-0.15, 0.23] | 1.04 | 0.15 | [-0.09, 0.40] | 1.17 |
|  | Ancestry PC5 | 0.06 | [-0.12, 0.24] | 1.06 | 0.10 | [-0.12, 0.33] | 1.11 |
|  | Ancestry PC6 | 0.16 | [-0.03, 0.35] | 1.17 | 0.10 | [-0.14, 0.33] | 1.10 |
|  | Ancestry PC7 | 0.13 | [-0.06, 0.32] | 1.14 | 0.02 | [-0.23, 0.27] | 1.02 |
|  | Ancestry PC8 | 0.08 | [-0.10, 0.26] | 1.08 | 0.20 | [-0.04, 0.43] | 1.22 |
|  | Ancestry PC9 | -0.02 | [-0.19, 0.16] | 0.98 | -0.03 | [-0.27, 0.20] | 0.97 |
|  | Ancestry PC10 | 0.02 | [-0.17, 0.20] | 1.02 | 0.11 | [-0.12, 0.34] | 1.11 |
| High risk  (*n*=171) | Intercept | -2.08*** | [-2.44, -1.73] | 0.12 |  |  |  |
|  | Depression PRS | 0.31** | [0.12, 0.50] | 1.36 |  |  |  |
|  | Externalizing PRS | -0.04 | [-0.23, 0.16] | 0.96 |  |  |  |
|  | Sex | 1.15*** | [0.77, 1.53] | 3.17 |  |  |  |
|  | Parental depression | 0.76*** | [0.35, 1.16] | 2.13 |  |  |  |
|  | Parental anxiety | -0.07 | [-0.55, 0.41] | 0.93 |  |  |  |
|  | Parental substance use | 0.48 | [-0.25, 1.21] | 1.61 |  |  |  |
|  | Parental antisocial | 0.13 | [-0.58, 0.84] | 1.14 |  |  |  |
|  | SES | -0.40*** | [-0.59, -0.21] | 0.67 |  |  |  |
|  | Ancestry PC1 | 0.20* | [0.00, 0.39] | 1.22 |  |  |  |
|  | Ancestry PC2 | -0.16 | [-0.36, 0.03] | 0.85 |  |  |  |
|  | Ancestry PC3 | -0.24* | [-0.43, -0.04] | 0.79 |  |  |  |
|  | Ancestry PC4 | -0.11 | [-0.34, 0.11] | 0.89 |  |  |  |
|  | Ancestry PC5 | -0.04 | [-0.25, 0.17] | 0.96 |  |  |  |
|  | Ancestry PC6 | 0.06 | [-0.15, 0.27] | 1.06 |  |  |  |
|  | Ancestry PC7 | 0.11 | [-0.12, 0.34] | 1.11 |  |  |  |
|  | Ancestry PC8 | -0.11 | [-0.32, 0.09] | 0.89 |  |  |  |
|  | Ancestry PC9 | 0.01 | [-0.20, 0.23] | 1.02 |  |  |  |
|  | Ancestry PC10 | -0.09 | [-0.30, 0.12] | 0.91 |  |  |  |

*Note.* ^*^*p*<.05, ^**^*p*<.01, ^***^*p*<.001. Sex: 0=female, 1=male. Parent psychopathology: 1=present in father and/or mother, 0=not present in either. OR: Odds Ratio. CI: 95% Confidence Interval. “PC” = Principal component.

Table S8. *Regression results internalizing outcomes using temperament trajectory groups and PRS (Reference group=Low Risk)*

|  | Withdrawal/Depression | | | | | | Anxiety/Depression | | | | | |
| --- | --- | --- | --- | --- | --- | --- | --- | --- | --- | --- | --- | --- |
| Term | B | SE | *p* | B | SE | *p* | B | SE | *p* | B | SE | *p* |
| (Intercept) | -0.31 | 0.06 | 0.000 | -0.41 | 0.06 | 0.000 | -0.07 | 0.06 | 0.544 | -0.20 | 0.06 | 0.009 |
| Overcontrolled | 0.36 | 0.09 | 0.000 | 0.16 | 0.10 | 0.426 | 0.21 | 0.09 | 0.141 | 0.15 | 0.09 | 0.329 |
| Low Affiliation | 0.28 | 0.08 | 0.000 | 0.16 | 0.08 | 0.213 | 0.26 | 0.07 | 0.009 | 0.20 | 0.07 | 0.047 |
| Undercontrolled | 0.08 | 0.09 | 0.804 | -0.02 | 0.09 | 0.891 | 0.28 | 0.09 | 0.016 | 0.17 | 0.09 | 0.252 |
| High Risk | 0.65 | 0.10 | 0.000 | 0.18 | 0.12 | 0.426 | 0.43 | 0.10 | 0.000 | 0.20 | 0.11 | 0.269 |
| Dep PRS | 0.02 | 0.03 | 0.518 | 0.01 | 0.03 | 0.632 | 0.08 | 0.03 | 0.008 | 0.06 | 0.03 | 0.029 |
| Ext PRS | 0.05 | 0.03 | 0.114 | 0.05 | 0.03 | 0.088 | -0.01 | 0.03 | 0.863 | 0.00 | 0.03 | 0.977 |
| Sex | 0.13 | 0.06 | 0.029 | 0.10 | 0.06 | 0.103 | -0.33 | 0.06 | 0.000 | -0.29 | 0.06 | 0.000 |
| Parental depression | 0.07 | 0.07 | 0.310 | 0.03 | 0.07 | 0.669 | 0.02 | 0.07 | 0.739 | -0.01 | 0.07 | 0.883 |
| Parental anxiety | 0.03 | 0.08 | 0.732 | 0.02 | 0.08 | 0.789 | 0.12 | 0.08 | 0.120 | 0.09 | 0.08 | 0.232 |
| Parental substance | 0.08 | 0.13 | 0.552 | 0.03 | 0.13 | 0.809 | -0.03 | 0.13 | 0.833 | -0.04 | 0.13 | 0.736 |
| Parental antisocial | 0.10 | 0.12 | 0.402 | 0.06 | 0.11 | 0.622 | -0.02 | 0.12 | 0.858 | -0.04 | 0.12 | 0.751 |
| SES | -0.05 | 0.03 | 0.124 | -0.04 | 0.03 | 0.137 | 0.02 | 0.03 | 0.559 | 0.02 | 0.03 | 0.511 |
| Ancestry PC1 | -0.04 | 0.03 | 0.245 | -0.04 | 0.03 | 0.215 | -0.03 | 0.03 | 0.323 | -0.03 | 0.03 | 0.293 |
| Ancestry PC2 | -0.02 | 0.03 | 0.470 | -0.01 | 0.03 | 0.629 | -0.05 | 0.03 | 0.082 | -0.05 | 0.03 | 0.079 |
| Ancestry PC3 | 0.02 | 0.03 | 0.633 | 0.01 | 0.03 | 0.708 | 0.00 | 0.03 | 0.983 | 0.00 | 0.03 | 0.876 |
| Ancestry PC4 | -0.02 | 0.03 | 0.606 | -0.01 | 0.03 | 0.718 | -0.04 | 0.03 | 0.220 | -0.04 | 0.03 | 0.272 |
| Ancestry PC5 | 0.01 | 0.03 | 0.850 | 0.00 | 0.03 | 0.915 | 0.00 | 0.03 | 0.882 | -0.01 | 0.03 | 0.760 |
| Ancestry PC6 | 0.05 | 0.03 | 0.148 | 0.04 | 0.03 | 0.230 | 0.02 | 0.03 | 0.460 | 0.02 | 0.03 | 0.548 |
| Ancestry PC7 | -0.04 | 0.03 | 0.154 | -0.04 | 0.03 | 0.213 | -0.03 | 0.03 | 0.310 | -0.03 | 0.03 | 0.293 |
| Ancestry PC8 | 0.02 | 0.03 | 0.568 | 0.01 | 0.03 | 0.674 | 0.02 | 0.03 | 0.421 | 0.02 | 0.03 | 0.593 |
| Ancestry PC9 | 0.01 | 0.03 | 0.835 | 0.00 | 0.03 | 0.872 | 0.06 | 0.03 | 0.069 | 0.05 | 0.03 | 0.115 |
| Ancestry PC10 | 0.02 | 0.03 | 0.613 | 0.02 | 0.03 | 0.530 | 0.01 | 0.03 | 0.638 | 0.02 | 0.03 | 0.574 |
| T1-T3 Control |  |  |  | 0.89 | 0.14 | 0.000 |  |  |  | 0.76 | 0.15 | 0.000 |

*Note.* “PC” = Principal component; T1-T3 control = Corresponding covariate for each outcome collected at the first to third waves of data collection (i.e., parent-report of child withdrawal/depression and child anxiety/depression); *p*-values for primary variables (i.e., temperament trajectory groups and PRS) have been adjusted using Benjamini and Hochberg (1995) method or false discovery rate (i.e., q-value).

Table S9. *Regression results predicting externalizing outcomes using temperament trajectory groups and PRS (Reference group=Low Risk)*

|  | Aggressive Behavior | | | | | | Delinquent Behavior | | | | | | Attention Problems | | | | | |
| --- | --- | --- | --- | --- | --- | --- | --- | --- | --- | --- | --- | --- | --- | --- | --- | --- | --- | --- |
| Term | B | SE | *p* | B | SE | *p* | B | SE | *p* | B | SE | *p* | B | SE | *p* | B | SE | *p* |
| (Intercept) | -0.14 | 0.06 | 0.084 | -0.21 | 0.06 | 0.000 | -0.38 | 0.06 | 0.000 | -0.42 | 0.06 | 0.000 | -0.28 | 0.06 | 0.000 | -0.34 | 0.06 | 0.000 |
| Overcontrolled | 0.07 | 0.09 | 0.725 | 0.07 | 0.09 | 0.734 | -0.07 | 0.09 | 0.605 | -0.07 | 0.09 | 0.610 | 0.02 | 0.09 | 0.888 | 0.01 | 0.09 | 0.915 |
| Low Affiliation | 0.27 | 0.07 | 0.000 | 0.22 | 0.07 | 0.019 | 0.25 | 0.07 | 0.000 | 0.22 | 0.07 | 0.012 | 0.29 | 0.07 | 0.000 | 0.24 | 0.08 | 0.007 |
| Undercontrolled | 0.55 | 0.09 | 0.000 | 0.42 | 0.09 | 0.000 | 0.51 | 0.09 | 0.000 | 0.42 | 0.10 | 0.000 | 0.61 | 0.09 | 0.000 | 0.46 | 0.10 | 0.000 |
| High Risk | 0.49 | 0.10 | 0.000 | 0.30 | 0.11 | 0.044 | 0.51 | 0.10 | 0.000 | 0.41 | 0.11 | 0.000 | 0.68 | 0.10 | 0.000 | 0.51 | 0.11 | 0.000 |
| Dep PRS | 0.06 | 0.03 | 0.030 | 0.06 | 0.03 | 0.034 | -0.04 | 0.03 | 0.191 | -0.04 | 0.03 | 0.211 | -0.01 | 0.03 | 0.668 | -0.01 | 0.03 | 0.765 |
| Ext PRS | 0.05 | 0.03 | 0.104 | 0.04 | 0.03 | 0.170 | 0.13 | 0.03 | 0.000 | 0.12 | 0.03 | 0.000 | 0.03 | 0.03 | 0.355 | 0.02 | 0.03 | 0.538 |
| Sex | -0.22 | 0.06 | 0.000 | -0.23 | 0.06 | 0.000 | 0.38 | 0.06 | 0.000 | 0.38 | 0.06 | 0.000 | -0.01 | 0.06 | 0.793 | -0.04 | 0.06 | 0.445 |
| Parental depression | 0.02 | 0.07 | 0.798 | 0.00 | 0.07 | 0.990 | -0.07 | 0.07 | 0.296 | -0.09 | 0.07 | 0.215 | 0.05 | 0.07 | 0.469 | 0.04 | 0.06 | 0.573 |
| Parental anxiety | 0.09 | 0.08 | 0.233 | 0.09 | 0.08 | 0.229 | 0.02 | 0.08 | 0.811 | 0.02 | 0.08 | 0.797 | 0.02 | 0.08 | 0.833 | 0.01 | 0.08 | 0.853 |
| Parental substance | -0.16 | 0.13 | 0.206 | -0.15 | 0.13 | 0.239 | 0.12 | 0.14 | 0.393 | 0.11 | 0.14 | 0.426 | 0.10 | 0.12 | 0.437 | 0.09 | 0.12 | 0.454 |
| Parental antisocial | 0.16 | 0.12 | 0.198 | 0.12 | 0.12 | 0.336 | 0.45 | 0.13 | 0.001 | 0.41 | 0.13 | 0.002 | 0.13 | 0.12 | 0.270 | 0.10 | 0.12 | 0.392 |
| SES | -0.07 | 0.03 | 0.026 | -0.05 | 0.03 | 0.105 | 0.02 | 0.03 | 0.600 | 0.03 | 0.03 | 0.318 | 0.01 | 0.03 | 0.677 | 0.03 | 0.03 | 0.364 |
| Ancestry PC1 | -0.02 | 0.03 | 0.530 | -0.02 | 0.03 | 0.520 | -0.01 | 0.03 | 0.709 | -0.01 | 0.03 | 0.658 | -0.04 | 0.03 | 0.141 | -0.05 | 0.03 | 0.112 |
| Ancestry PC2 | -0.01 | 0.03 | 0.829 | 0.00 | 0.03 | 0.984 | -0.01 | 0.03 | 0.662 | -0.01 | 0.03 | 0.754 | -0.02 | 0.03 | 0.495 | -0.02 | 0.03 | 0.555 |
| Ancestry PC3 | 0.01 | 0.03 | 0.729 | 0.01 | 0.03 | 0.762 | 0.04 | 0.03 | 0.196 | 0.04 | 0.03 | 0.243 | 0.03 | 0.03 | 0.309 | 0.03 | 0.03 | 0.334 |
| Ancestry PC4 | -0.01 | 0.03 | 0.748 | -0.01 | 0.03 | 0.856 | 0.01 | 0.03 | 0.664 | 0.02 | 0.03 | 0.611 | 0.00 | 0.03 | 0.970 | 0.01 | 0.03 | 0.860 |
| Ancestry PC5 | -0.01 | 0.03 | 0.839 | -0.01 | 0.03 | 0.815 | -0.03 | 0.03 | 0.302 | -0.03 | 0.03 | 0.297 | -0.04 | 0.03 | 0.252 | -0.04 | 0.03 | 0.248 |
| Ancestry PC6 | 0.03 | 0.03 | 0.418 | 0.02 | 0.03 | 0.452 | 0.05 | 0.03 | 0.086 | 0.05 | 0.03 | 0.097 | 0.05 | 0.03 | 0.119 | 0.04 | 0.03 | 0.169 |
| Ancestry PC7 | -0.03 | 0.03 | 0.308 | -0.03 | 0.03 | 0.284 | -0.04 | 0.04 | 0.270 | -0.04 | 0.04 | 0.280 | -0.04 | 0.03 | 0.241 | -0.04 | 0.03 | 0.224 |
| Ancestry PC8 | 0.05 | 0.03 | 0.098 | 0.05 | 0.03 | 0.098 | 0.06 | 0.03 | 0.060 | 0.06 | 0.03 | 0.049 | 0.05 | 0.03 | 0.132 | 0.05 | 0.03 | 0.145 |
| Ancestry PC9 | 0.02 | 0.03 | 0.604 | 0.01 | 0.03 | 0.690 | -0.05 | 0.03 | 0.074 | -0.06 | 0.03 | 0.070 | -0.01 | 0.03 | 0.809 | -0.01 | 0.03 | 0.815 |
| Ancestry PC10 | 0.00 | 0.03 | 0.909 | 0.01 | 0.03 | 0.837 | 0.01 | 0.03 | 0.650 | 0.01 | 0.03 | 0.662 | 0.02 | 0.03 | 0.551 | 0.02 | 0.03 | 0.541 |
| T1-T3 Control |  |  |  | 0.47 | 0.13 | 0.000 |  |  |  | 0.69 | 0.28 | 0.012 |  |  |  | 0.34 | 0.12 | 0.004 |

*Note.* “PC” = Principal component; T1-T3 control = Corresponding covariate for each outcome collected at the first to third waves of data collection (i.e., parent-report of child withdrawal/depression and child anxiety/depression); *p*-values for primary variables (i.e., temperament trajectory groups and PRS) have been adjusted using Benjamini and Hochberg (1995) method or false discovery rate (i.e., q-value).

Table S10. *Regression results predicting internalizing outcomes using temperament trajectory groups and PRS (Reference group=High Risk)*

|  | Withdrawal/Depression | | | | | | Anxiety/Depression | | | | | |
| --- | --- | --- | --- | --- | --- | --- | --- | --- | --- | --- | --- | --- |
| Term | B | SE | *p* | B | SE | *p* | B | SE | *p* | B | SE | *p* |
| (Intercept) | 0.34 | 0.10 | 0.009 | -0.22 | 0.13 | 0.426 | 0.36 | 0.10 | 0.000 | 0.01 | 0.12 | 0.983 |
| Overcontrolled | -0.57 | 0.12 | 0.000 | -0.21 | 0.13 | 0.426 | -0.16 | 0.11 | 0.449 | -0.03 | 0.12 | 0.943 |
| Low Affiliation | -0.37 | 0.10 | 0.000 | -0.02 | 0.12 | 0.891 | -0.18 | 0.10 | 0.269 | 0.00 | 0.11 | 0.983 |
| Undercontrolled | -0.29 | 0.12 | 0.149 | -0.02 | 0.13 | 0.891 | -0.22 | 0.12 | 0.269 | -0.05 | 0.12 | 0.930 |
| High Risk | -0.65 | 0.10 | 0.000 | -0.18 | 0.12 | 0.426 | -0.43 | 0.10 | 0.000 | -0.20 | 0.11 | 0.269 |
| Dep PRS | 0.02 | 0.03 | 0.518 | 0.01 | 0.03 | 0.632 | 0.08 | 0.03 | 0.007 | 0.06 | 0.03 | 0.029 |
| Ext PRS | 0.05 | 0.03 | 0.114 | 0.05 | 0.03 | 0.088 | -0.01 | 0.03 | 0.863 | 0.00 | 0.03 | 0.977 |
| Sex | 0.13 | 0.06 | 0.029 | 0.10 | 0.06 | 0.103 | -0.33 | 0.06 | 0.000 | -0.29 | 0.06 | 0.000 |
| Parental depression | 0.07 | 0.07 | 0.310 | 0.03 | 0.07 | 0.669 | 0.02 | 0.07 | 0.739 | -0.01 | 0.07 | 0.883 |
| Parental anxiety | 0.03 | 0.08 | 0.732 | 0.02 | 0.08 | 0.789 | 0.12 | 0.08 | 0.120 | 0.09 | 0.08 | 0.232 |
| Parental substance | 0.08 | 0.13 | 0.552 | 0.03 | 0.13 | 0.809 | -0.03 | 0.13 | 0.833 | -0.04 | 0.13 | 0.736 |
| Parental antisocial | 0.10 | 0.12 | 0.402 | 0.06 | 0.11 | 0.622 | -0.02 | 0.12 | 0.858 | -0.04 | 0.12 | 0.751 |
| SES | -0.05 | 0.03 | 0.124 | -0.04 | 0.03 | 0.137 | 0.02 | 0.03 | 0.559 | 0.02 | 0.03 | 0.511 |
| Ancestry PC1 | -0.04 | 0.03 | 0.245 | -0.04 | 0.03 | 0.215 | -0.03 | 0.03 | 0.323 | -0.03 | 0.03 | 0.293 |
| Ancestry PC2 | -0.02 | 0.03 | 0.470 | -0.01 | 0.03 | 0.629 | -0.05 | 0.03 | 0.082 | -0.05 | 0.03 | 0.079 |
| Ancestry PC3 | 0.02 | 0.03 | 0.633 | 0.01 | 0.03 | 0.708 | 0.00 | 0.03 | 0.983 | 0.00 | 0.03 | 0.876 |
| Ancestry PC4 | -0.02 | 0.03 | 0.606 | -0.01 | 0.03 | 0.718 | -0.04 | 0.03 | 0.220 | -0.04 | 0.03 | 0.272 |
| Ancestry PC5 | 0.01 | 0.03 | 0.850 | 0.00 | 0.03 | 0.915 | 0.00 | 0.03 | 0.882 | -0.01 | 0.03 | 0.760 |
| Ancestry PC6 | 0.05 | 0.03 | 0.148 | 0.04 | 0.03 | 0.230 | 0.02 | 0.03 | 0.460 | 0.02 | 0.03 | 0.548 |
| Ancestry PC7 | -0.04 | 0.03 | 0.154 | -0.04 | 0.03 | 0.213 | -0.03 | 0.03 | 0.310 | -0.03 | 0.03 | 0.293 |
| Ancestry PC8 | 0.02 | 0.03 | 0.568 | 0.01 | 0.03 | 0.674 | 0.02 | 0.03 | 0.421 | 0.02 | 0.03 | 0.593 |
| Ancestry PC9 | 0.01 | 0.03 | 0.835 | 0.00 | 0.03 | 0.872 | 0.06 | 0.03 | 0.069 | 0.05 | 0.03 | 0.115 |
| Ancestry PC10 | 0.02 | 0.03 | 0.613 | 0.02 | 0.03 | 0.530 | 0.01 | 0.03 | 0.638 | 0.02 | 0.03 | 0.574 |
| T1-T3 Control |  |  |  | 0.89 | 0.14 | 0.000 |  |  |  | 0.76 | 0.15 | 0.000 |

*Note.* “PC” = Principal component; T1-T3 control = Corresponding covariate for each outcome collected at the first to third waves of data collection (i.e., parent-report of child withdrawal/depression and child anxiety/depression); *p*-values for primary variables (i.e., temperament trajectory groups and PRS) have been adjusted using Benjamini and Hochberg (1995) method or false discovery rate (i.e., q-value).

Table S11. *Regression results predicting externalizing outcomes using temperament trajectory groups and PRS (Reference group=High Risk)*

|  | Aggressive Behavior | | | | | | Delinquent Behavior | | | | | | Attention Problems | | | | | |
| --- | --- | --- | --- | --- | --- | --- | --- | --- | --- | --- | --- | --- | --- | --- | --- | --- | --- | --- |
| Term | B | SE | *p* | B | SE | *p* | B | SE | *p* | B | SE | *p* | B | SE | *p* | B | SE | *p* |
| (Intercept) | 0.35 | 0.35 | 0.007 | 0.09 | 0.12 | 0.734 | 0.13 | 0.10 | 0.412 | -0.01 | 0.12 | 0.933 | 0.40 | 0.09 | 0.000 | 0.17 | 0.13 | 0.605 |
| Overcontrolled | 0.07 | 0.07 | 0.788 | 0.12 | 0.11 | 0.558 | 0.00 | 0.12 | 0.988 | 0.01 | 0.12 | 0.933 | -0.07 | 0.11 | 0.534 | -0.06 | 0.11 | 0.807 |
| Low Affiliation | -0.21 | -0.21 | 0.139 | -0.08 | 0.11 | 0.725 | -0.26 | 0.10 | 0.052 | -0.19 | 0.11 | 0.193 | -0.39 | 0.10 | 0.000 | -0.27 | 0.11 | 0.083 |
| Undercontrolled | -0.42 | -0.42 | 0.000 | -0.23 | 0.13 | 0.242 | -0.58 | 0.12 | 0.000 | -0.48 | 0.13 | 0.000 | -0.65 | 0.12 | 0.000 | -0.50 | 0.13 | 0.000 |
| High Risk | -0.49 | -0.49 | 0.000 | -0.30 | 0.11 | 0.044 | -0.51 | 0.10 | 0.000 | -0.41 | 0.11 | 0.000 | -0.68 | 0.10 | 0.000 | -0.51 | 0.11 | 0.000 |
| Dep PRS | 0.06 | 0.06 | 0.030 | 0.06 | 0.03 | 0.034 | -0.04 | 0.03 | 0.191 | -0.04 | 0.03 | 0.211 | -0.01 | 0.03 | 0.668 | -0.01 | 0.03 | 0.765 |
| Ext PRS | 0.05 | 0.05 | 0.104 | 0.04 | 0.03 | 0.170 | 0.13 | 0.03 | 0.000 | 0.12 | 0.03 | 0.000 | 0.03 | 0.03 | 0.355 | 0.02 | 0.03 | 0.538 |
| Sex | -0.22 | -0.22 | 0.000 | -0.23 | 0.06 | 0.000 | 0.38 | 0.06 | 0.000 | 0.38 | 0.06 | 0.000 | -0.01 | 0.06 | 0.793 | -0.04 | 0.06 | 0.445 |
| Parental depression | 0.02 | 0.02 | 0.798 | 0.00 | 0.07 | 0.990 | -0.07 | 0.07 | 0.296 | -0.09 | 0.07 | 0.215 | 0.05 | 0.07 | 0.469 | 0.04 | 0.06 | 0.573 |
| Parental anxiety | 0.09 | 0.09 | 0.233 | 0.09 | 0.08 | 0.229 | 0.02 | 0.08 | 0.811 | 0.02 | 0.08 | 0.797 | 0.02 | 0.08 | 0.833 | 0.01 | 0.08 | 0.853 |
| Parental substance | -0.16 | -0.16 | 0.206 | -0.15 | 0.13 | 0.239 | 0.12 | 0.14 | 0.393 | 0.11 | 0.14 | 0.426 | 0.10 | 0.12 | 0.437 | 0.09 | 0.12 | 0.454 |
| Parental antisocial | 0.16 | 0.16 | 0.198 | 0.12 | 0.12 | 0.336 | 0.45 | 0.13 | 0.001 | 0.41 | 0.13 | 0.002 | 0.13 | 0.12 | 0.270 | 0.10 | 0.12 | 0.392 |
| SES | -0.07 | -0.07 | 0.026 | -0.05 | 0.03 | 0.105 | 0.02 | 0.03 | 0.600 | 0.03 | 0.03 | 0.318 | 0.01 | 0.03 | 0.677 | 0.03 | 0.03 | 0.364 |
| Ancestry PC1 | -0.02 | -0.02 | 0.530 | -0.02 | 0.03 | 0.520 | -0.01 | 0.03 | 0.709 | -0.01 | 0.03 | 0.658 | -0.04 | 0.03 | 0.141 | -0.05 | 0.03 | 0.112 |
| Ancestry PC2 | -0.01 | -0.01 | 0.829 | 0.00 | 0.03 | 0.984 | -0.01 | 0.03 | 0.662 | -0.01 | 0.03 | 0.754 | -0.02 | 0.03 | 0.495 | -0.02 | 0.03 | 0.555 |
| Ancestry PC3 | 0.01 | 0.01 | 0.729 | 0.01 | 0.03 | 0.762 | 0.04 | 0.03 | 0.196 | 0.04 | 0.03 | 0.243 | 0.03 | 0.03 | 0.309 | 0.03 | 0.03 | 0.334 |
| Ancestry PC4 | -0.01 | -0.01 | 0.748 | -0.01 | 0.03 | 0.856 | 0.01 | 0.03 | 0.664 | 0.02 | 0.03 | 0.611 | 0.00 | 0.03 | 0.970 | 0.01 | 0.03 | 0.860 |
| Ancestry PC5 | -0.01 | -0.01 | 0.839 | -0.01 | 0.03 | 0.815 | -0.03 | 0.03 | 0.302 | -0.03 | 0.03 | 0.297 | -0.04 | 0.03 | 0.252 | -0.04 | 0.03 | 0.248 |
| Ancestry PC6 | 0.03 | 0.03 | 0.418 | 0.02 | 0.03 | 0.452 | 0.05 | 0.03 | 0.086 | 0.05 | 0.03 | 0.097 | 0.05 | 0.03 | 0.119 | 0.04 | 0.03 | 0.169 |
| Ancestry PC7 | -0.03 | -0.03 | 0.308 | -0.03 | 0.03 | 0.284 | -0.04 | 0.04 | 0.270 | -0.04 | 0.04 | 0.280 | -0.04 | 0.03 | 0.241 | -0.04 | 0.03 | 0.224 |
| Ancestry PC8 | 0.05 | 0.05 | 0.098 | 0.05 | 0.03 | 0.098 | 0.06 | 0.03 | 0.060 | 0.06 | 0.03 | 0.049 | 0.05 | 0.03 | 0.132 | 0.05 | 0.03 | 0.145 |
| Ancestry PC9 | 0.02 | 0.02 | 0.604 | 0.01 | 0.03 | 0.690 | -0.05 | 0.03 | 0.074 | -0.06 | 0.03 | 0.070 | -0.01 | 0.03 | 0.809 | -0.01 | 0.03 | 0.815 |
| Ancestry PC10 | 0.00 | 0.00 | 0.909 | 0.01 | 0.03 | 0.837 | 0.01 | 0.03 | 0.650 | 0.01 | 0.03 | 0.662 | 0.02 | 0.03 | 0.551 | 0.02 | 0.03 | 0.541 |
| T1-T3 Control |  |  |  | 0.47 | 0.13 | 0.000 |  |  |  | 0.69 | 0.28 | 0.012 |  |  |  | 0.34 | 0.12 | 0.004 |

*Note.* “PC” = Principal component; T1-T3 control = Corresponding covariate for each outcome collected at the first to third waves of data collection (i.e., parent-report of child withdrawal/depression and child anxiety/depression); *p*-values for primary variables (i.e., temperament trajectory groups and PRS) have been adjusted using Benjamini and Hochberg (1995) method or false discovery rate (i.e., q-value).


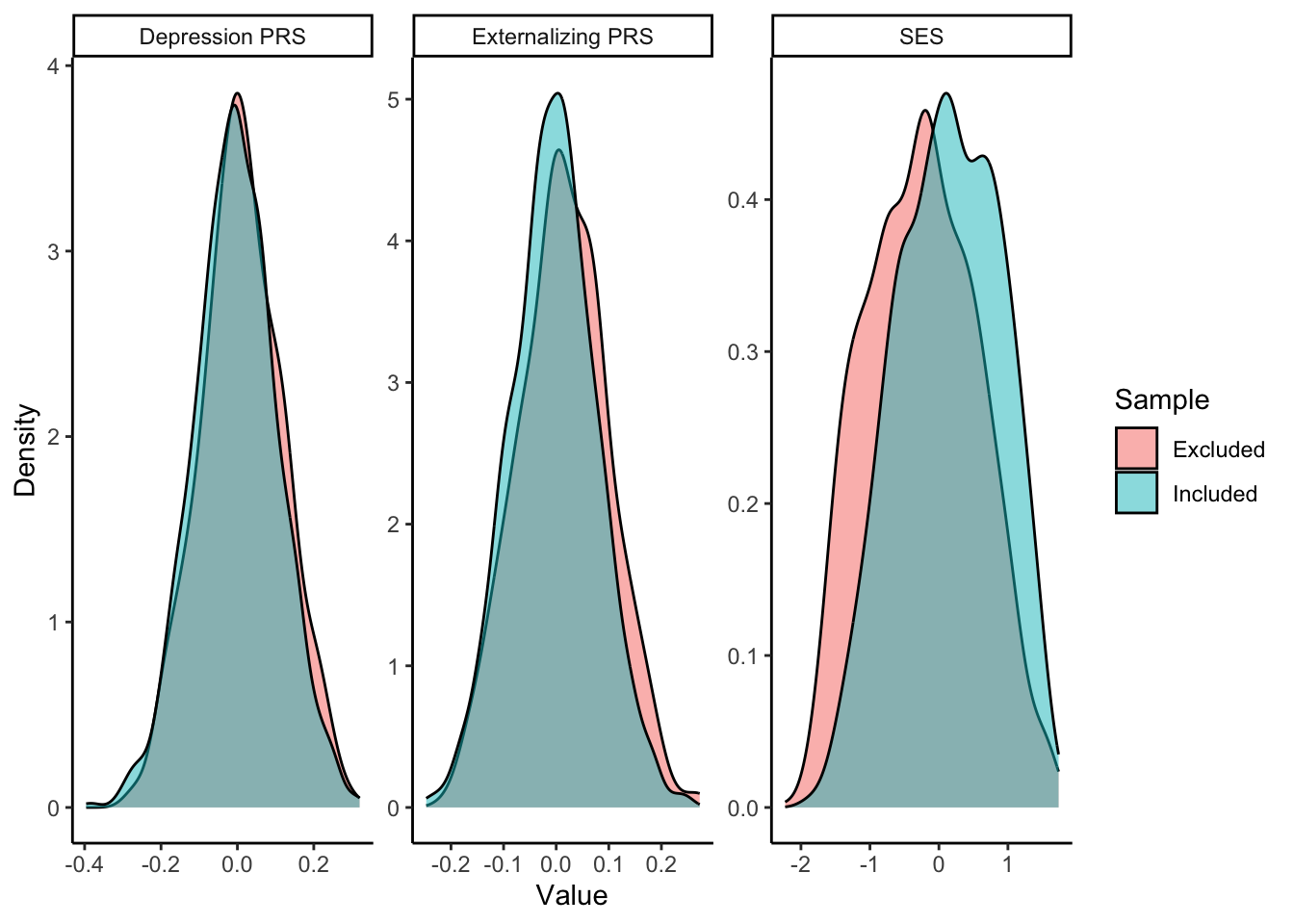


Figure S1. *Density plots of the numeric predictors in the included vs. excluded samples*


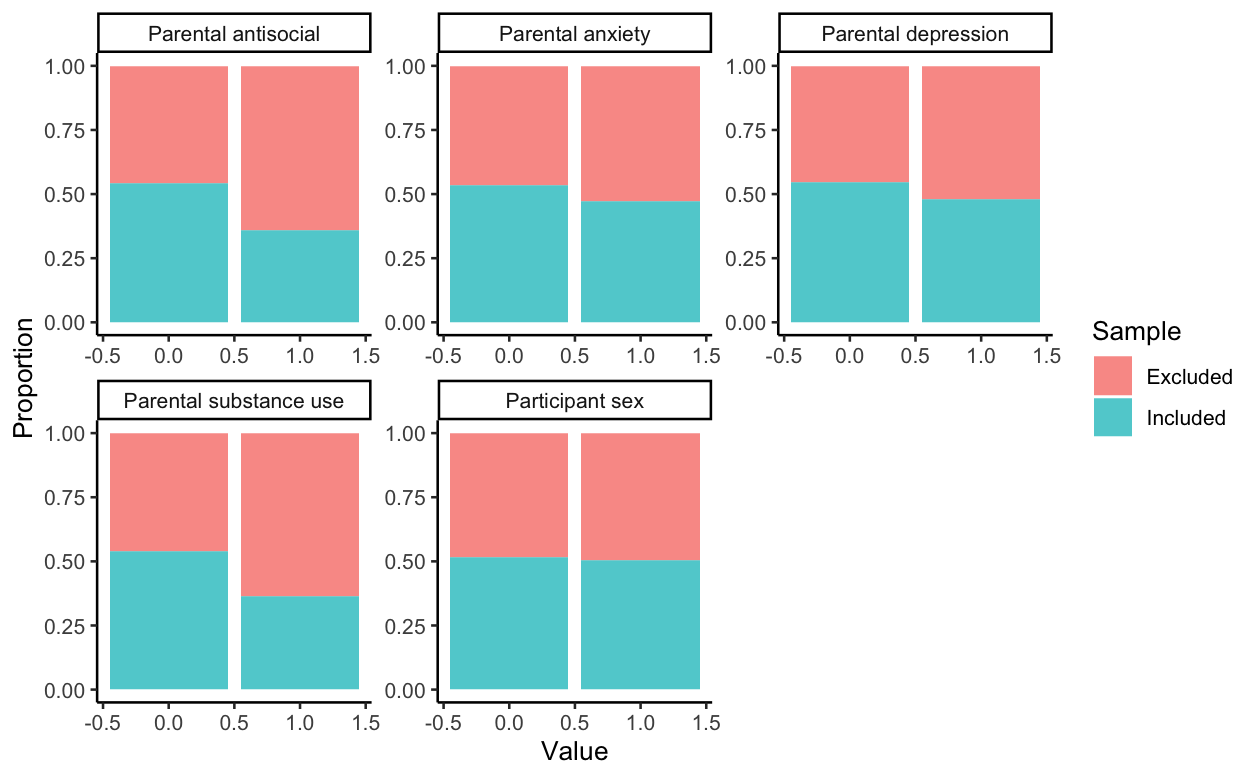


Figure S2. *Stacked bar charts of the binary covariates in the included vs. excluded samples*

*
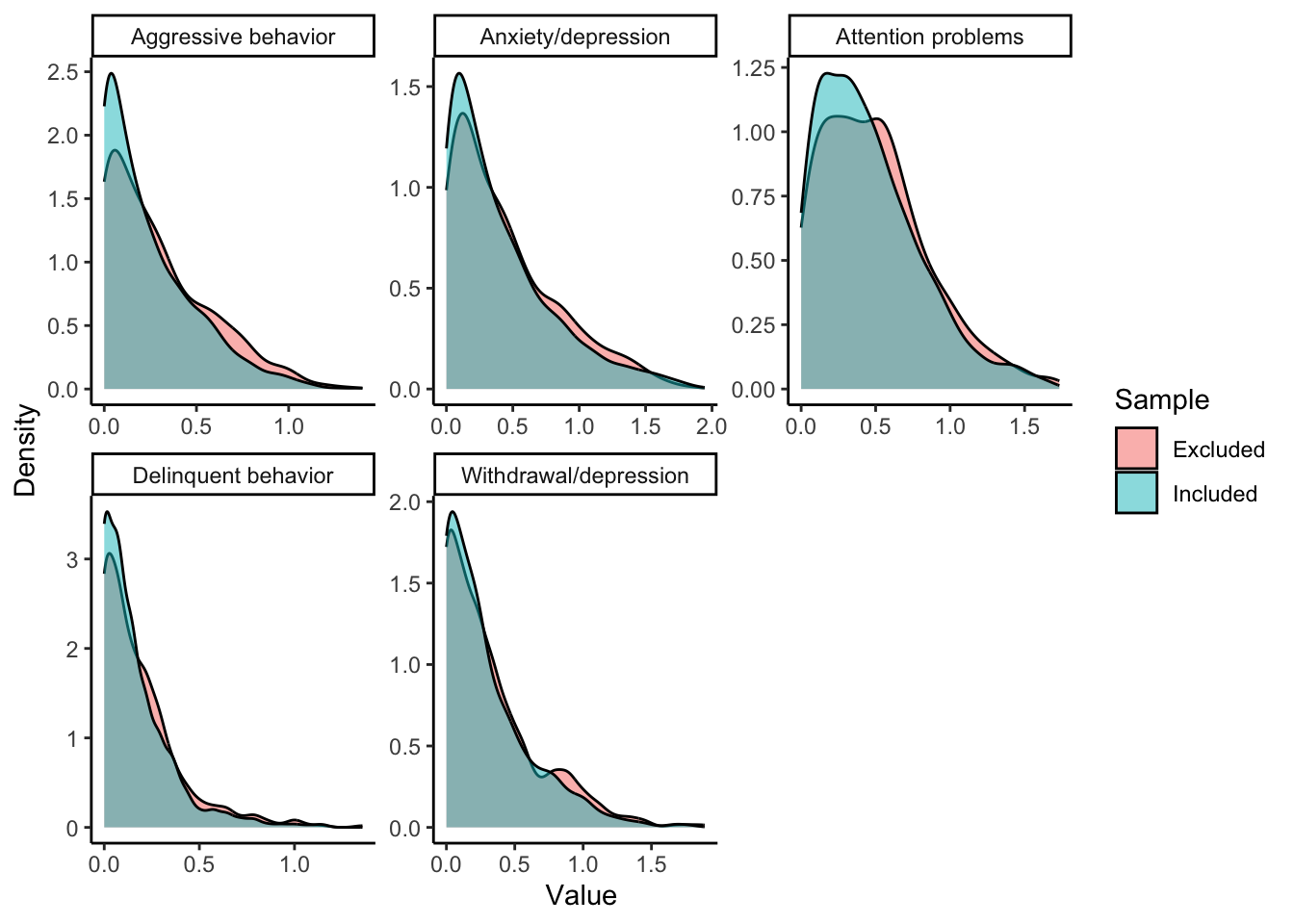
*

Figure S3. *Density plots of the numeric outcomes as a function of the included vs. excluded samples*

*
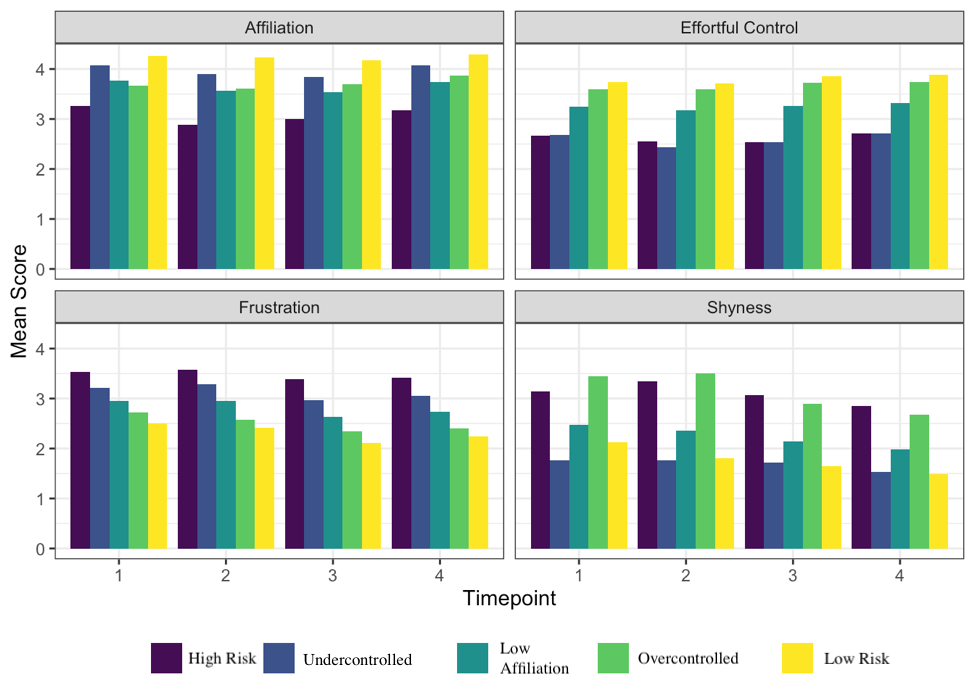
*

Figure S4. Differences in temperament domains by trajectory group. Timepoint 1=T1; Timepoint 2= T3; Timepoint 3=T4; Timepoint 4=T5.


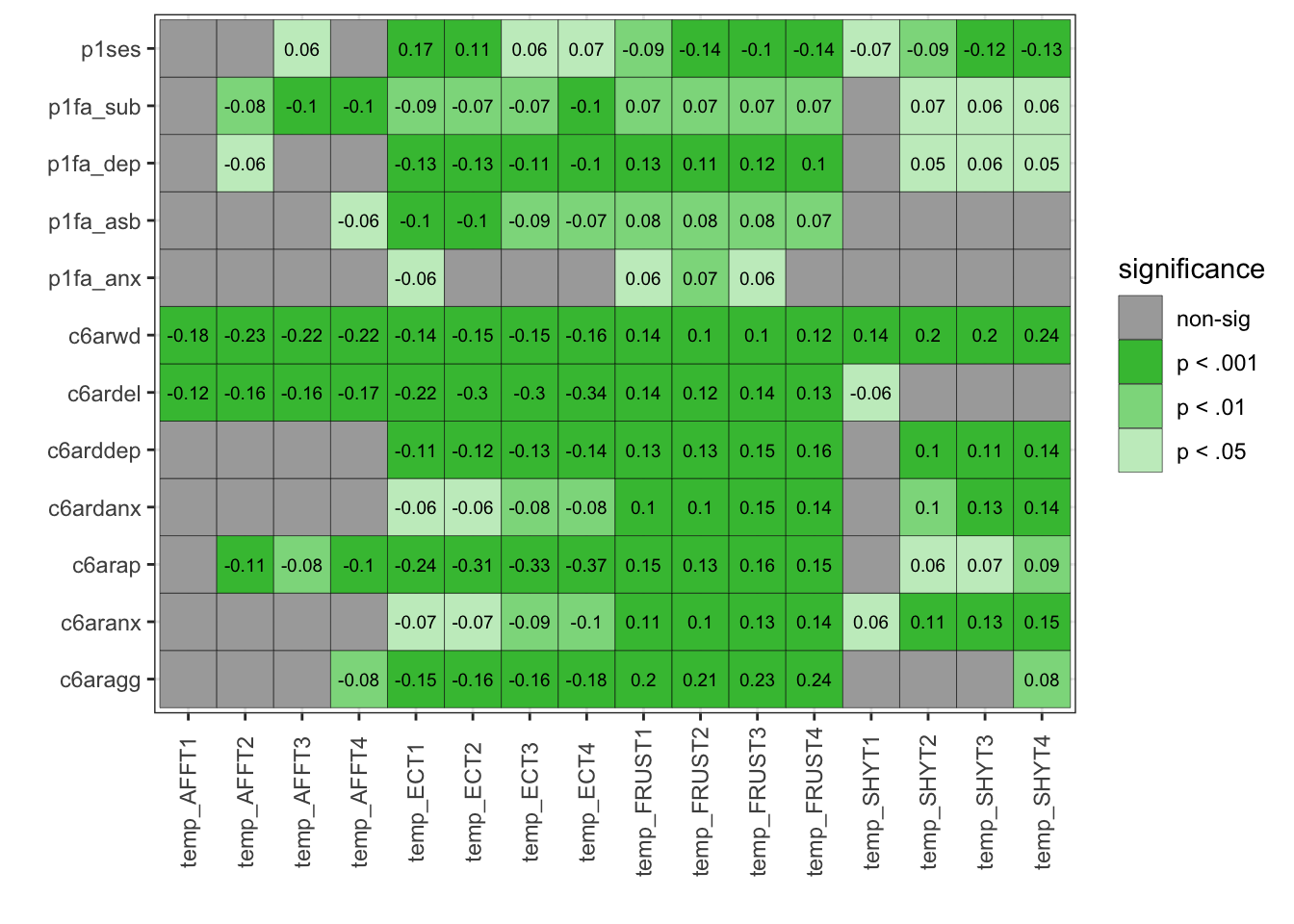


Figure S5. Zero-order correlations among temperament dimensions, adulthood psychopathology, and parental psychopathology. AFF: Affiliation. EC: Effortful Control. FRUS: Frustration. Shy: Shyness. P1ses: Familial SES. P1fa_sub: Parental substance use. P1fa_dep: Parental depression. P1fa_asb: parental antisocial behavior. P1fa_anx: parental anxiety. c6arwd: Withdrawn / Depressed subscale. c6aranx: Anxious / Depressed subscale. c6aragg: Aggressive Behavior subscale. c6ardel: Delinquent Behavior subscale. c6arap: Attention Problems subscale. c6arddep: DSM-oriented depression scale. c6ardanx: DSM-oriented anxiety scale.


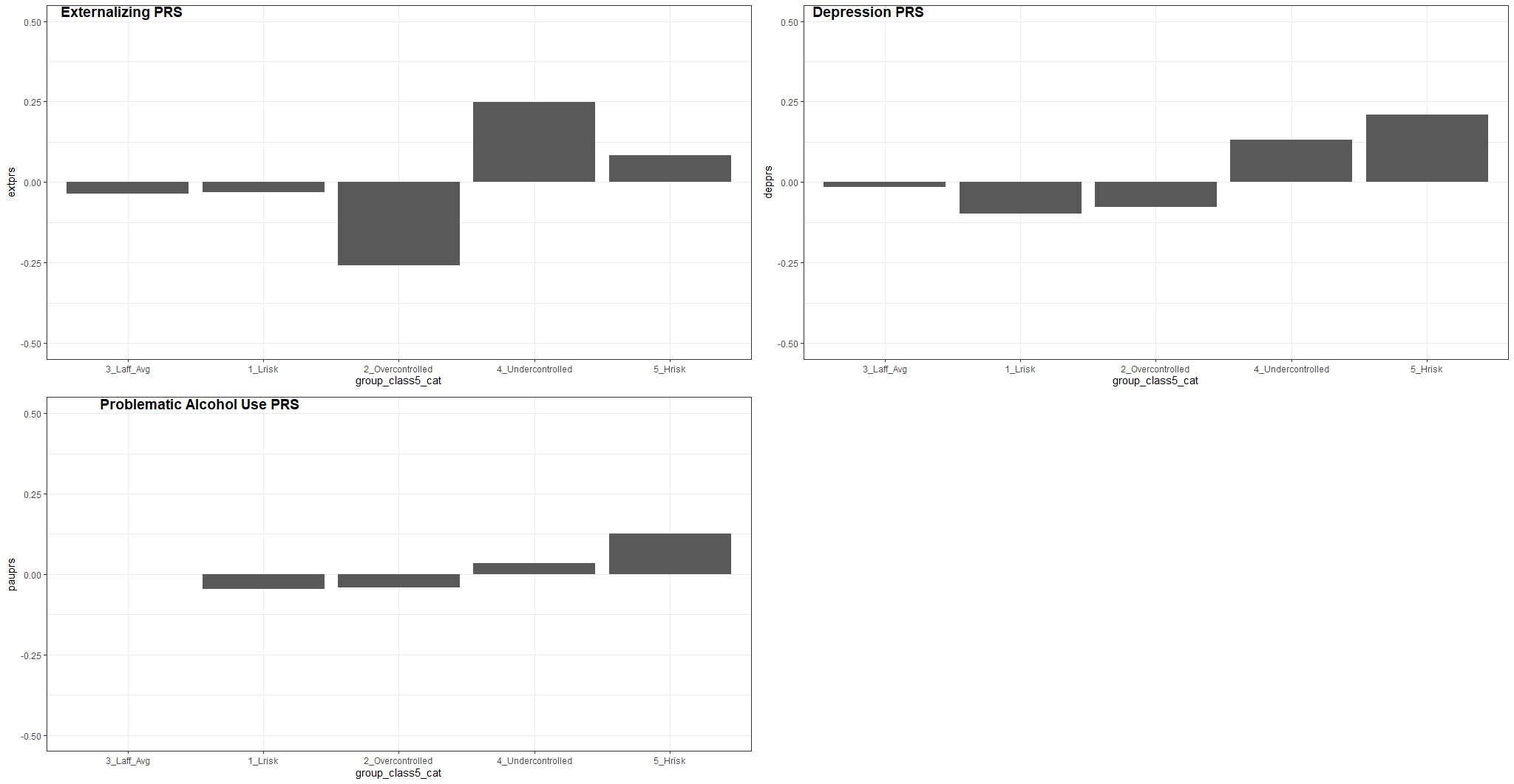


Figure S6. Means of Polygenic Risk Scores by Temperament Trajectory Groups. Polygenic risk scores are standardized.


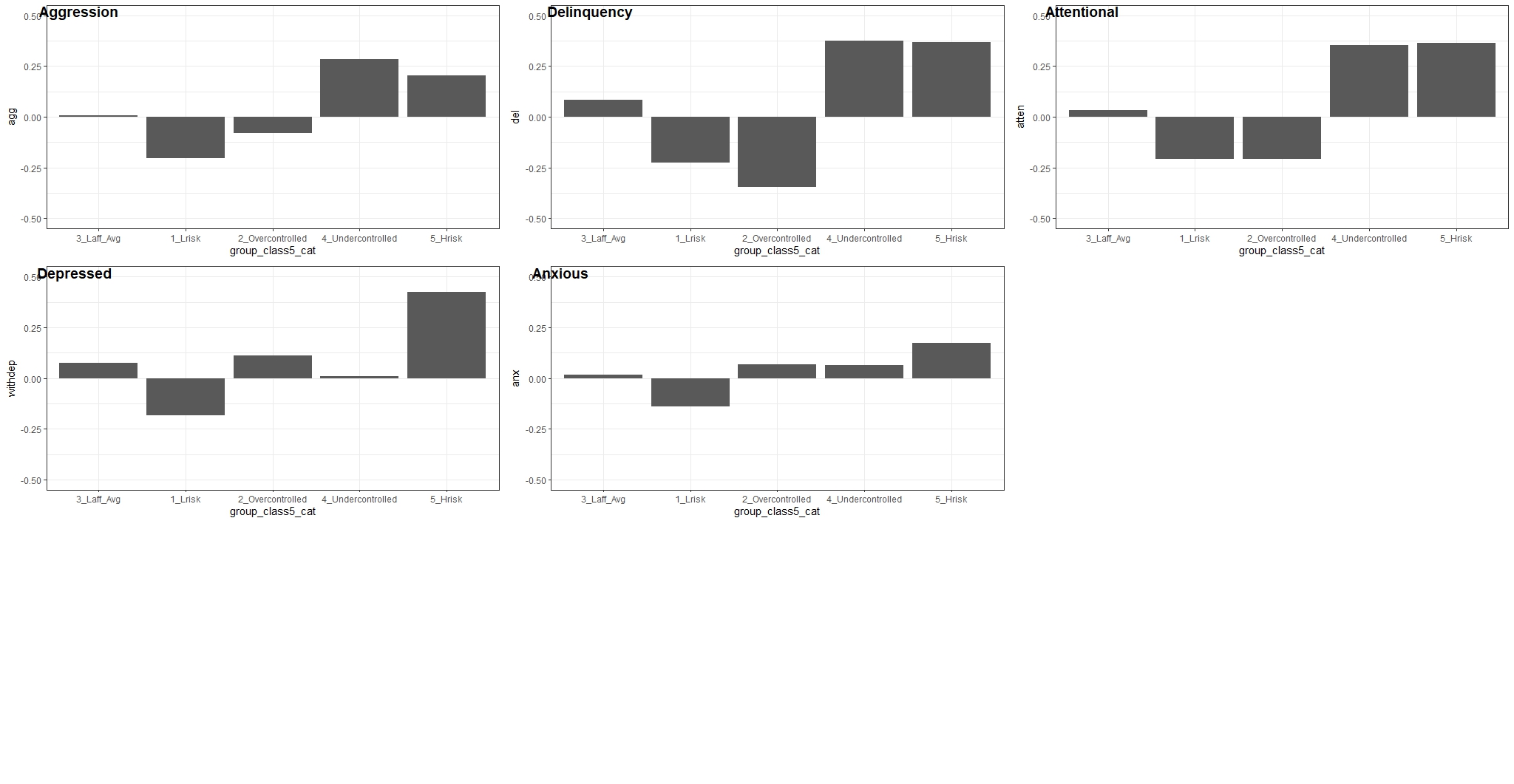


Figure S7. Means of Externalizing and Internalizing Problems by Temperament Trajectory Groups. All outcomes are standardized.
